# A robust machine learning framework for predicting contact angle in nano-assisted chemical EOR

**DOI:** 10.1038/s41598-026-48016-1

**Published:** 2026-05-08

**Authors:** Youssef E. Kandiel, Omar Mahmoud, Ahmed Farid Ibrahim

**Affiliations:** 1 Department of Petroleum and Energy Engineering, School of Sciences and Engineering, American University in Cairo (AUC), Cairo, Egypt; 2 Department of Chemical and Petroleum Engineering, College of Engineering and Physical Sciences, Khalifa University, 127788 , Abu Dhabi, United Arab Emirates; 3 Department of Petroleum Engineering and Geosciences, King Fahd University of Petroleum & Minerals, 31261 Dhahran, Saudi Arabia; 4 Center for Integrative Petroleum Research, King Fahd University of Petroleum & Minerals, 31261 Dhahran, Saudi Arabia

**Keywords:** Machine learning, Nanoparticles, Wettability, Enhanced oil recovery, SHAP, Rock-type optimization, Energy science and technology, Engineering, Materials science, Mathematics and computing

## Abstract

Wettability alteration through nano-assisted chemical Enhanced Oil Recovery (nano-cEOR) critically governs capillary pressure and multiphase flow in porous media. Contact angle (CA) quantifies wettability, yet experimental measurement remains costly, inconsistent, and operationally impractical, creating nanofluid screening bottlenecks. Existing machine learning (ML) models lack nanoparticle (NP)-specific descriptors essential for nano-cEOR physics. This study introduces a comprehensive ML framework predicting CA from 418 experimental data points, integrating NP-specific descriptors (type, concentration, size, functionalization), fluid properties, rock mineralogy, and reservoir conditions. Six algorithms were benchmarked across independent training, testing, and validation datasets with hyperparameter optimization and tenfold nested cross-validation. Multi-method sensitivity analysis integrating Sobol indices, SHAP values, and partial dependence plots identified operational thresholds and lithology-specific strategies. XGBoost Regressor (XGBR) achieved superior validation performance: R^2^ = 0.953, RMSE = 9.24°, MAE = 5.87°, and near-zero prediction bias (− 0.04°). Sensitivity analysis identified a minimum permeability of 0.1 mD for nano-cEOR viability, an optimal salinity window of 30,000–80,000 ppm, and synergistic NP-to-chemical ratios of 1:1 to 1.5:1. Rock-type analysis revealed lithology-dependent formulation inversion, with optimal NPs shifting between carbonates (ZrO_2_, TiO_2_) and sandstones (Fe_3_O_4_, CuO). These findings advance nanofluid screening capability, support reservoir-specific formulation design, and provide decision-support guidelines for field-scale nano-cEOR deployment.

**Supplementary Information:**

The online version contains supplementary material available at 10.1038/s41598-026-48016-1.

## Introduction

The global energy demand continues to necessitate the maximized recovery of hydrocarbon resources from existing reservoirs. However, conventional recovery methods are fundamentally inefficient, typically leaving 50% to 60%^1–4^, and in some formations as high as 80%^5^, of the original oil in place immobile as residual oil saturation. This vast, unrecovered volume constitutes the primary target for all modern Enhanced Oil Recovery (EOR) techniques. The challenge is particularly acute in carbonate reservoirs, which hold approximately 60% of global oil reserves^3,5^. These formations exhibit naturally oil-wet to intermediate-wet conditions, where crude oil adheres strongly to rock surfaces, creating low or negative capillary forces that prevent spontaneous water imbibition^2,6^. This condition causes injected water to channel through fracture networks—bypassing the oil-bearing matrix—and leads to early breakthrough at production wells, leaving the bulk of oil resources trapped^3,5^. Recovery from waterflooding alone can be as low as 5–10% in oil-wet carbonates^6^, confirming that wettability is the single most dominant parameter governing oil mobilization and the primary target for EOR intervention^7^.

The central engineering strategy to address this challenge is Wettability Alteration (WA); shifting rock preference from oil-wet (hydrophobic) to water-wet (hydrophilic), quantified by the fluid–fluid-solid Contact Angle (CA)^8^. While traditional chemical EOR (cEOR) has long employed surfactants to modify interfacial properties^9^, recent advancements have introduced nanoparticles (NPs) as potent WA-agents through two distinct mechanisms: (1) structural disjoining pressure—where NPs self-assemble at the three-phase contact line to physically detach adhered oil films^2,6,10^, and (2) irreversible adsorption onto rock substrates, forming a nanoscale hydrophilic layer that masks the original oil-wet mineralogy^3,11^. This nano-assisted EOR (nEOR) approach achieves dramatic CA shifts from strongly oil-wet (130–140°) to strongly water-wet (30–40°)^8,12^.

This nano-assisted approach represents a sophisticated hybrid EOR method, particularly when NPs are functionalized with chemical agents. In this synergistic model, the NPs core act as a high-surface-area delivery vehicle, concentrating chemical agents (e.g., surfactants, polymers) directly at the rock-fluid interface. Simultaneously, the NPs themselves introduce potent physical mechanisms (like disjoining pressure) that are absent in traditional chemical floods. Exemplary systems include iron oxide (Fe₃O₄) NPs functionalized with a hydrophilic polymer (e.g., Ethylenediaminetetraacetic acid, EDTA) or an ionic surfactant (e.g., Sodium Lauryl Sulfate, SLS)^6^. Further studies have shown synergistic effects by combining titanium oxide (TiO₂) NPs with natural surfactants (e.g., from Tribulus terrestris extract)^3^. Advanced material design has also produced core–shell nanocomposites, such as Fe₃O₄ coated with gelatin biopolymer (Fe₃O₄@gelatin), which exhibit exceptional performance in modifying oil-wet dolomite and calcite surfaces to a strongly water-wet state^2^.

Despite the well-documented efficacy of nano-assisted chemical EOR (nano-cEOR), a critical knowledge gap persists. The distinct physical mechanisms of nano-cEOR, and the performance of these synergistic formulations, are inherently dependent on the specific properties of the NPs themselves, including their chemical type, concentration, size, surface coating, and chemical additive. While the efficacy of these functionalized and hybrid NPs is well-established in laboratory experiments, the complex interplay between the NPs core, the chemical functionalization, and the specific crude-oil/brine/rock system remains incompletely characterized.

More fundamentally, predicting wettability under specific formulation and reservoir conditions is fraught with experimental and practical challenges. Although the CA is the definitive parameter for quantifying wettability, robust measurement under reservoir-representative conditions is a profound bottleneck. Goniometric and Amott-based techniques are time-consuming, expensive, highly sensitive to fluid properties, and yield inconsistent results due to surface heterogeneity and environmental sensitivity^13–15^. Under ultra-low interfacial tension (IFT) conditions, even fundamental measurements fail as oil droplets may spread too thinly for CA determination^16^. Computational approaches, such as Pore Network Models and molecular simulations, also face severe limitations due to computational cost, the need for extensive manual intervention, and inability to capture all relevant complexities of natural rock-fluid systems^17–19^.

These converging bottlenecks have motivated a shift toward data-driven and machine learning (ML) methodologies. Recent advances in applied ML demonstrate exceptional success in predicting wettability and CA across analogous domains, with models achieving high predictive accuracy (R^2^ values ranging from 0.86 to 0.99)^17,20–23^. This state-of-the-art includes diverse applications, from image-based wettability classification^24,25^ and relative permeability prediction^19,26^ to parametric regression for EOR percentage^27,28^ and dynamic^29^ or static CA^30–32^. Other studies have extended these models to complex subsurface environments involving shale mineralogy and hydrogen storage^33–36^. Ensemble tree-based algorithms,particularly Random Forest (RF, Extreme Gradient Boost (XGBoost, and CatBoost have emerged as the state-of-the-art for parametric prediction problems, consistently demonstrating top-tier performance^17,20,21,28,31,32,37–39^. These models are particularly powerful because they can (i) handle high-dimensional, non-linear interdependencies, (ii) perform robustly on the small, sparse datasets (e.g., 120 to 800 + data points) typical of experimental work^28,31,32^, and (iii) provide interpretability through SHAP (Shapley Additive Explanations) analysis^32,39^. The most advanced precedents employ physics-informed machine learning (PIML), embedding physical constraints directly into the model to ensure physically realistic predictions^39^.

However, a critical and previously unaddressed gap exists: while robust ML methodologies and extensive literature precedents for wettability prediction now exist, the current state-of-the-art models are physically incomplete for nano-cEOR. Existing high-performance models are parameterized only for bulk material properties^17,22^, operational conditions (pressure, temperature, salinity)^21,32^, and surface morphology^20^. None of the existing models incorporate the NPs-specific descriptors essential to capture the fundamental physics of nano-cEOR—namely, NPs type, concentration, size, and surface functionalization, which directly govern the structural disjoining pressure mechanism^2^. Consequently, existing models cannot be applied to design or predict wettability for nanofluid formulations in EOR systems.

A clear distinction exists between the present work and previously published ML studies on wettability and CA prediction. Sena et al.^38^ developed an XGBR model for water CA on solid polymer surfaces using cross-validation only, without external validation, and reported no sensitivity-derived operational outputs. Vo Thanh^39^ introduced a physics-informed ANN for hydrogen–rock wettability prediction in underground hydrogen storage, but without nanoparticle descriptors or multi-lithology stratification. Wu et al.^32^ predicted shale–alkane–brine CA at reservoir conditions using ML, focusing on geological mineralogy descriptors rather than NP formulation parameters. Hajibolouri and Shafiei^33^ addressed wettability in hydrogen storage systems using gradient boosting, but without chemical additive descriptors or NP concentration features. Collectively, these studies establish high predictive accuracy for their respective domains but remain inapplicable to nano-cEOR design: none incorporate NP-specific descriptors (type, concentration, size, functionalization), none perform rock-type stratified sensitivity analysis to derive lithology-specific formulation guidelines, and none provide a framework covering both carbonate and sandstone reservoirs simultaneously. The present study explicitly fills this gap by constructing a multi-lithology, NP-descriptor-inclusive ML framework with integrated Sobol–SHAP–PDP sensitivity analysis, validated on an independent held-out dataset and benchmarked against the full existing literature (Supplementary Material S2, Table 2).

Hence, the present work introduces a ML framework for nano-cEOR, uniquely integrating both established predictors; such as fluid and rock properties, and operational parameters with a new set of NPs-specific features including NPs type, concentration, size, and surface functionalization. This comprehensive approach is built on a robust dataset compiled from peer-reviewed experimental nano-cEOR studies, ensuring broad representativeness and reliability. The framework undergoes rigorous validation and benchmarking across a range of advanced ML algorithms including tree-based models and neural network architectures to identify the most accurate and robust predictive models. Furthermore, advanced interpretability techniques such as SHAP analysis and feature importance mapping are employed to elucidate the underlying physical and chemical factors that drive wettability alteration in nEOR systems. Ultimately, this study delivers a validated, interpretable, and user-oriented predictive tool that enables rapid and cost-effective screening and optimization of nanofluid formulations for real-world, field-scale EOR deployment, significantly reducing experimental burden for initial nanofluid screening and formulation optimization.

This study is structured as follows. First, the experimental dataset is described, including curation protocol, data sources, and the statistical applicability domain of the resulting models (Section "Dataset curation and description"). Second, the feature engineering and selection rationale is presented, documenting the physical basis for each input descriptor (Section "Feature engineering and selection"). Third, the complete preprocessing workflow—including feature transformation, outlier management, imputation strategy, categorical encoding, and cross-validation protocol—is detailed (Section "Data preprocessing, processing, and preparation"). Fourth, the six ML architectures and hyperparameter optimization procedure are described (Sections "Machine learning model architectures"–"Hyperparameter optimization process"). Fifth, model performance is compared across training, testing, and validation splits using comprehensive statistical metrics and residual diagnostics (Section "Comparative performance of ML models"–"Model generalization and residual diagnostics"). Sixth, global and local sensitivity analyses (Sobol, SHAP, PDP, CDF stratification) are presented to derive operational thresholds and lithology-specific guidelines (Section "Comprehensive sensitivity analysis"). Finally, results are interpreted in the context of nano-cEOR physics, advantages and limitations of the framework are discussed, field deployment considerations are outlined, and recommendations for future research are provided (Sections "Discussion" and "Summary and conclusions").

## Methodology and data

### Dataset curation and description

The foundation of any robust ML model is a comprehensive and high-quality dataset. For this study, a comprehensive dataset of 418 experimental data points was meticulously curated, comprising 408 data points from peer-reviewed literature published between 2013 and 2025, supplemented by 10 data points from our own experimental work (Supplementary Materials S3). The experimental work for CA measurements performed using the captive drop method to quantify WA before and after NPs treatment using drop shape analysis software.This dataset represents a significant contribution in its own right, aggregating hundreds of disparate, time-consuming, and expensive experiments conducted in laboratories worldwide. This aggregation enables a large-scale meta-analysis and modeling effort that would be impossible for a single research group to perform experimentally. The dataset represented in Table 1, captures a wide range of nanofluid formulations, chemical environments, and reservoir conditions pertinent to modern nano-cEOR operations. Furthermore, by providing transparent and detailed documentation of the entire data sources (Supplementary Materials S3), the dataset facilitates reproducibility and supports duplication by interested peers, thereby strengthening the reliability and broader impact of subsequent research built upon these findings.

**Table 1:** Representative literature sources used for dataset curation.

Reference	Por. (%)	Perm. (mD)	Salinity (ppm)	NPs	NPs Conc. (wt%)	Chemical Additive	Additive Conc. (wt%)	Temp. (°C)	CA (°)
Mousavi et al.^40^	28.4	1.5—2	0	SiO_2_	2	Alcohols / Solvents	20	80	61
Giraldo et al.^41^	20	2.19	0	Al_2_O_3_	0.01	Nonionic Surfactant	1	Ambient	104
Hendraningrat et al.^42^	15.2	13	30,000	SiO_2_	0.01	None	None	Ambient	40
Hendraningrat et al.^43^	16.4	97	30,000	SiO_2_	0.05	None	None	Ambient	38.8
Ehtesabi et al.^44^	23.7	84	5000	TiO_2_	0.01	None	None	Ambient	90
Hendraningrat and Torsæter^45^	14.7	330	30,000	Al_2_O_3_	0.05	Nonionic Polymer	0.1	Ambient	28.6
Hendraningrat and Torsæter^45^	14.9	118	30,000	TiO_2_	0.05	Nonionic Polymer	0.1	Ambient	21.6
Hendraningrat and Torsæter^46^	19	300	30,000	SiO_2_	0.05	None	None	Ambient	26
Joonaki and Ghanaatian^47^	17.4	110.4	25,000	Fe_3_O_4_	0.3	Alcohols / Solvents	N/A	Ambient	98
Bayat et al.^48^	42.7	2340	3000	SiO_2_	0.005	None	None	60	18
Mohammadi et al.^49^	17.3	0.807	180,000	Al_2_O_3_	0.1	None	None	Ambient	54
Hendraningrat and Torsæter^50^	16	230	30,000	Al_2_O_3_	0.05	Nonionic Polymer	1	Ambient	28.6
Zhang et al.^51^	28.6	54,730	2500	SiO_2_	0.277	Nonionic Surfactant	N/A	25	25
Nwidee et al.^52^	19.5	370	0	NiO	0.05	Cationic Surfactant	0.5	22	48.3
Nwidee et al.^52^	19.5	318	0	ZrO_2_	0.05	Nonionic Surfactant	0.5	22	60
Li et al.^53^	14.1	0.61	30,000	SiO_2_	1	None	None	60	38
Saha et al.^54^	26.5	1002	4445	SiO_2_	0.5	Biosurfactant	0.5	70	18.8
Wang et al.^55^	13	5	30,000	SiO_2_	1	Biosurfactant	0.004	Ambient	28
Safaei et al.^56^	13.9	2.39	0	Fe_3_O_4_	0.4	Cationic Surfactant	0.1	Ambient	62
Amrouche et al.^57^	28.1	7.5	41,158	MgO	0.0025	None	0	Ambient	55
Experimental	15.2	4.53	30,000	MgO	0.5	SDS	0.5	33	6.5

To ensure dataset quality and minimize the influence of measurement artifacts, a systematic data quality control protocol was applied during curation. Inclusion criteria required that each data point: (1) report a numerically quantified CA value (in degrees) measured under defined fluid/rock/temperature conditions; (2) specify at minimum the NP type, NP concentration, rock type, and brine salinity; and (3) originate from a peer-reviewed experimental study. Data points reporting only qualitative wettability assessments, or lacking primary experimental conditions, were excluded. The dataset encompasses multiple measurement methodologies, including captive drop, sessile drop, and tilted plate approaches, reflecting real-world inter-laboratory diversity. This heterogeneity represents an acknowledged source of measurement uncertainty goniometric technique, surface preparation protocol, and equilibration time vary across studies^15^ and is treated as natural variability rather than systematic error. To mitigate the influence of outlying measurements arising from experimental anomalies, percentile-based trimming was applied (data below the 1st or above the 99th percentile excluded), and residual diagnostic analysis confirmed that no systematic bias attributable to a single publication dominates model predictions. The compiled dataset encompasses studies from twelve countries and multiple independent research groups, providing broad representativeness across experimental protocols. This inter-laboratory diversity, while introducing uncertainty, simultaneously enhances the generalizability of the resulting ML models to conditions beyond any single laboratory’s experimental scope.

### Feature engineering and selection

The selection of appropriate input and output variables is critical to the model’s success. The target variable (output) for this study is the experimentally measured CA (θ), in degrees, which serves as the definitive proxy for wettability.

The input features were selected based on the known physical and chemical drivers of wettability identified in the literature^15,58–60^. These represent the key parameters an engineer can control or must account for when designing a nanofluid flood. The primary features include:

- **Nanofluid properties**
- NPs Type: A categorical feature describing the NPs chemistry (e.g., SiO_2_, Al_2_O_3_, Fe_2_O_3_, ZrO_2_, MgO)^59,60^. The NPs used in the dataset are categorized as: None (NP0), SiO_2_ (NP1), Al_2_O_3_ (NP2), TiO_2_ (NP3), Fe_3_O_4_ (NP4), NiO (NP5), ZrO_2_ (NP6), CuO (NP7), CN (NP8), MgO (NP9), and ZnO (NP10).
- NPs Concentration (NPs Conc.): A continuous feature, typically in weight percent (wt%), known to have a dose-dependent effect on WA^59,60^.
- NPs Size: A continuous feature, typically in nm, known to have a secondary effect on WA^59,60^.
- Nanofluid Viscosity: A continuous feature, typically in Centipoise (cP), known to have a secondary effect on WA^59^.
- **Chemical environment**
- Brine Salinity: A continuous feature (ppm or wt%) known to be a critical, highly sensitive parameter^15,59,60^. Salinity range of the base fluid represented in Table 2.
- Surfactant/Polymer Type and Concentration: Categorical and continuous features, as NPs are often co-injected with other chemicals^59^. The chemical additives used in the dataset are very diverse and categorized as: Anionic Surfactant (chem1), Cationic Surfactant (chem2), Nonionic Surfactant (chem3), Biosurfactant (chem4), Alcohols / Solvents (chem5), Anionic Polymer (chem6), and Nonionic Polymer (chem7).
- **Reservoir conditions**
- Temperature: A continuous feature (°C), as EOR operations occur at elevated reservoir temperatures^15,59^.
- **Fluid properties**
- Oil Viscosity: A continuous feature (cP), which is a proxy component in the oil^58,59^. The curated dataset ranges Table 2.
- Oil Specific Gravity (SG)/API: A continuous feature where SG is unitless and API typically in degree (°). The dataset have a wide diverse range of SG; and API.
- **Rock properties**
- Rock Type: A categorical feature (e.g., Carbonate, Sandstone) that defines the surface chemistry of the reservoir^59^. The rock used in the dataset categorized as: Limestone (R1), Sandstone (R2), Glass (R3), Dolomite (R4), and Carbonate (R5).
- Rock Petrophysical parameters: A continuous feature where porosity expressed in percentage (%), and permeability in millidarcy (mD). which is a proxy for the rock that affecting its wettability^58,59^.

**Table 2:** Statistical description of input features and output target in the curated dataset (N = 418).

Parameter	Count	Mean (μ)	Std Dev (σ)	Min	25% (Q1​)	50% (Median)	75% (Q3​)	Max
Porosity (%)	418	31.79	12.06	11.4	15.18	20.7	42.8	52.2
Permeability (mD)	418	509.84	823.59	0.13	9.75	150	2050	2400
Salinity (ppm)	418	20,031	15,821	0	3000	3000	30,000	180,000
NPs Size (nm)	418	22.81	25.1	0	0	15	40	93
NPs Conc. (wt%)	418	0.046	0.088	0	0	0.005	0.05	1.5
Additive Conc. (wt%)	418	0.093	0.241	0	0	0	0.1	1
Viscosity (cP)	418	1.28	0.89	0.353	0.999	1.001	1.28	11
Oil Viscosity (cP)	418	48.72	158.6	0.94	5.1	21.8	38	240
Oil SG	418	0.851	0.062	0.67	0.826	0.861	0.88	0.94
API Gravity	418	33.41	12.15	18	21.8	31.97	33.7	70.6
Temp. (°C)	418	38.54	21.3	15	15	26	50	90
CA (°)	418	79.45	39.62	6.5	31.18	71	114.8	180

The dataset were categorized into continuous (NPs Conc., NPs Size, Nanofluid Viscosity, Chemical Additive Conc., Salinity, Temp., Oil Viscosity, Oil SG/API, Porosity, Permeability, and CA), and categorical variables (NPs Type, Chemical Additive, Sample State, and Rock Type). A statistical summary of the continuous key variables in the dataset is presented in Table 2**.** This table defines the applicability domain of the resulting models; the models are expected to be accurate for predictions within the “Min” and “Max” ranges of these features.

**Note:** All Categorical parameters are excluded (NPs type, Chemical Additive type, Sample State, and Rock Type).

Exploratory analysis revealed non-linear interaction patterns, multimodal distributions, and log-normal skewness across experimental parameters. These topological complexities—including non-monotonic salinity-CA dependencies and discrete NPs stability clusters; demonstrate the inadequacy of linear correlation metrics, justifying the implementation of advanced ensemble and deep learning architectures for nano-cEOR modeling.

### Data preprocessing, processing, and preparation

To ensure the development of a robust and generalized ML framework for CA prediction, the raw experimental dataset underwent a rigorous multi-stage preprocessing workflow. This workflow was designed to address common challenges in nano-cEOR data, including high dimensionality, heterogeneous feature distributions, and measurement noise^35^. The preprocessing pipeline consisted of five key phases: feature transformation, outlier management, imputation, encoding/scaling, and Data spliting and Cross-Validation (CV).

#### Feature transformation and distributional correction

Key numerical features (permeability, salinity, NPs size, fluid viscosities) exhibited log-normal distributions with heavy right tails^61^. Natural logarithmic transformation (ln(x)) was applied to these variables to mitigate extreme value leverage, reduce skewness, and stabilize variance, facilitating more effective pattern recognition^62^.

#### Outlier detection and data cleaning

To prevent model bias from measurement artifacts, outliers were managed using percentile-based trimming: data points below the 1st or above the 99th percentile were excluded, eliminating gross deviations while preserving natural geological variability^63^.

#### Imputation and categorical encoding

Missing continuous values were imputed using median strategy, which is robust to outliers and appropriate for right-skewed distributions typical of petrophysical and nanofluid parameters^64–66^. For the NPs size feature specifically, the 25th percentile value of 0 nm in Table 2 reflects a proportion of data points for which in the source publication a chemical additive (modifier) is recognized as main treatment (cEOR) which justfy the purpose of addressing nano-cEOR rather than any other EOR approach. The potential for imputation bias was assessed by comparing SHAP attribution for NPs size between imputed and non-imputed subsets; the observed divergence between Sobol S1 (rank 4) and SHAP magnitude (rank 3) for NPs size is partly attributable to this imputation artifact, as median imputation compresses the variance of this feature and inflates SHAP contributions at the imputed value. Future datasets with complete NPs size characterization would be expected to sharpen the Sobol first-order index for this parameter. Nominal categorical variables (NPs Type, Sample State, Rock Type, Chemical Additive) were converted to numerical vectors via one-hot encoding (OHE). OHE was preferred over feature hashing and target encoding for the following reasons: (1) tree-based ensemble algorithms (XGBoost, RF, GBR) handle sparse binary feature matrices natively and efficiently without distance-metric assumptions that make OHE problematic for linear models; (2) target encoding introduces risk of target leakage within the cross-validation inner loop, as the encoding values are derived from the target variable; (3) OHE preserves full interpretability of categorical SHAP contributions, which is essential for the lithology-stratified sensitivity analysis central to this study. With 11 NP types generating 11 binary columns and 7 chemical additive categories generating 7 binary columns, the total encoded feature dimensionality is 36 (25 OHE + 11 continuous), which is well within the capacity of tree-based models for a dataset of 418 observations. It is acknowledged that minority categories (CN nanoparticles: 3 samples; ZnO: 5 samples) are underrepresented in the OHE space, and predictions for these categories should be interpreted with greater uncertainty.

#### Feature scaling and normalization

Right-skewed features (Permeability, Nanofluid Viscosity, Oil Viscosity) underwent logarithmic transformation: ln(x) for strictly positive values and ln(1 + x) for features with potential zeros (Salinity, NPs Size), mitigating extreme values and improving distribution symmetry. Following transformation, all continuous features and the target variable (CA) were Min–Max normalized to [0–1] (Eq. 1, Supplementary Material S1), ensuring equal contribution during optimization and preventing large-magnitude variables from dominating^67,68^.

#### Data splitting and cross validation

To evaluate model generalization and mitigate selection bias, the preprocessed dataset was partitioned into training (75%, 314 points), validation (12%, 50 points), and testing (13%, 54 points) sets for model fitting, hyperparameter tuning, and final unbiased performance assessment, respectively (Supplementary Materials S4). A tenfold nested CV scheme was implemented on the training set, with an inner loop for hyperparameter tuning and an outer loop for generalization error estimation. To address the potential for data leakage (within-publication data could appear in both training and evaluation folds) a Group-based Nested Cross-Validation (CV) strategy was implemented. In this setup, the ‘Publication Index’ was used as the grouping variable, ensuring that data points originating from the same study must be in the same fold. This approach provides a rigorous and conservative estimate of the generalization gap, as it simulates model performance on entirely unseen experimental environments reflects true predictive capability on unseen conditions^38,69^. This design is particularly important for practical EOR applications, where the deployed model must generalize to new nanofluid systems and reservoir environments not represented in its training history. Furthermore, five source publications Nwidee et al.^52^, Yekeen et al.^70^, Safaei et al.^56^, Tuok et al.^71^, and Amrouche et al.^57^ were entirely withheld from the training phase and reserved exclusively for independent testing and validation. These five studies were deliberately selected because, collectively, they span the full experimental domain of the dataset: NP types across the complete encoded range (NP0 through NP10), varying NP concentrations and particle sizes, both primary mineral types (sandstone and carbonate, including limestone and dolomite substrates), and the complete temperature spectrum represented in the dataset (ambient to 90 °C). This holdout strategy ensures that the reported model performance reflects true predictive capability on experimental environments the model has never encountered.

**Fig. 1:**
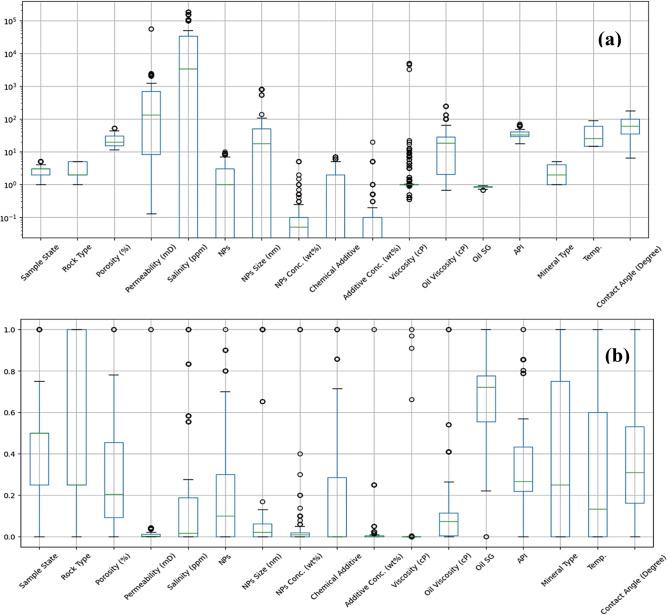
Box plot distribution of experimental parameters across the dataset, **a**) Raw data, **b**) Normalized data.

Figure 1 compares input feature distributions before and after preprocessing. Figure 1a (Raw Data) reveals substantial heterogeneity with parameters spanning multiple magnitude orders; Permeability and Salinity exhibit log-normal characteristics with heavy right tails typical of reservoir properties. Figure 1b (Normalized Data) demonstrates Min–Max normalization efficacy, mapping all features to [0–1] and preventing high-magnitude features (e.g., Viscosity ~ 5000 cP) from dominating loss function optimization. However, box plots reveal persistent skewness in NPs Concentration and Salinity (compressed IQRs, extended whiskers), confirming the necessity of preceding logarithmic transformation and outlier trimming to mitigate extreme value leverage^62,67^.

To investigate input parameter influence on CA, Spearman’s correlation coefficient (R) was calculated using Eq. 2 (Supplementary Material S1).

**Fig. 2:**
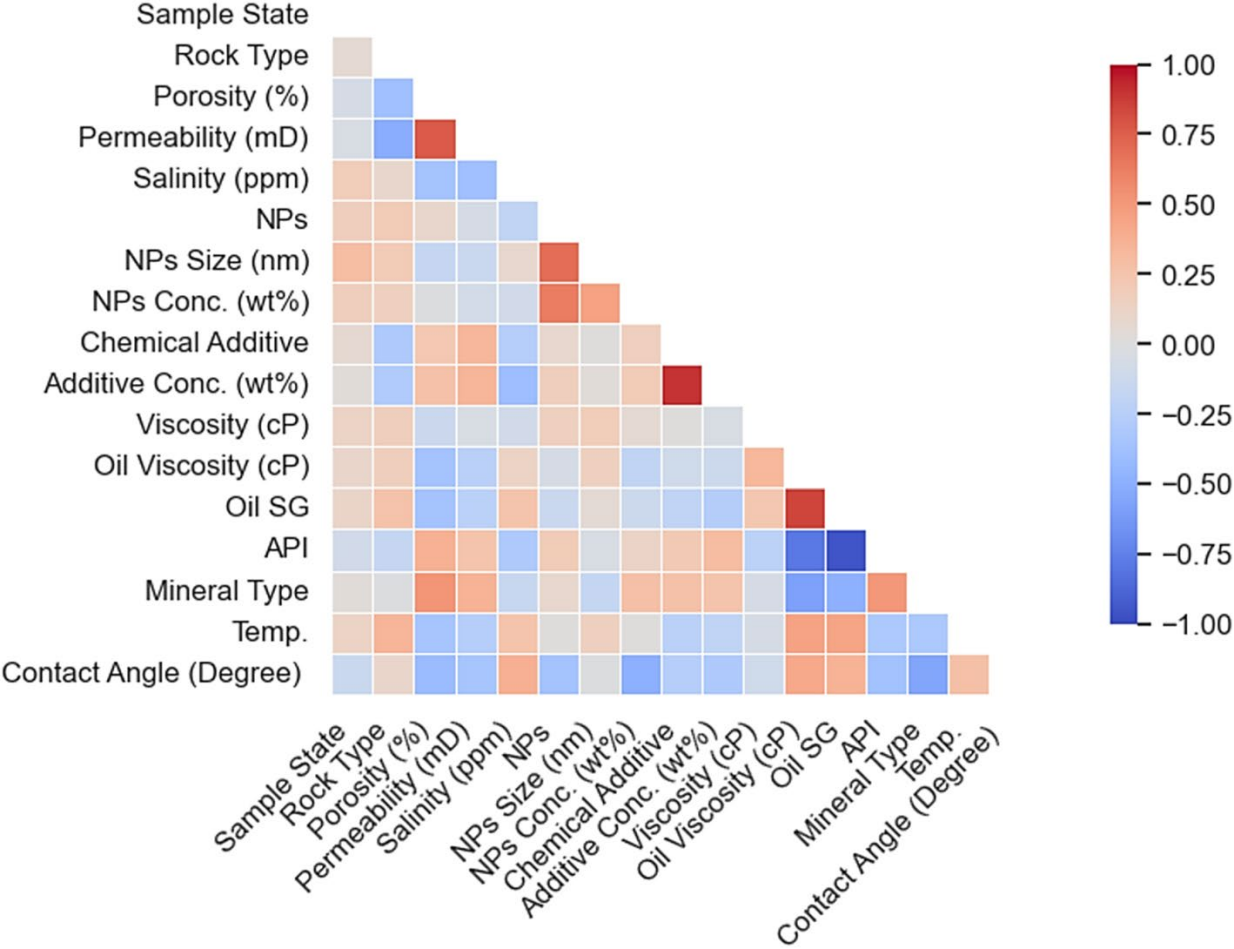
Pearson correlation matrix depicting linear relationships and multicollinearity among reservoir, fluid, NPs, and experimental variables versus CA measurements.

Figure 2 presents the Pearson correlation matrix, revealing linear dependencies and multicollinearity among experimental variables and CA. The matrix shows pronounced negative correlation (r≈ − 1.0) between oil SG and API gravity due to their inverse definitions, raising caution for linear modeling but posing no issue for tree-based ensembles^61^. CA exhibits moderate positive correlation with oil SG and strong negative correlation with API, confirming that heavier oils (higher SG, lower API) promote oil-wet surfaces and increased CA. Both NPs and chemical additive concentrations show moderate negative correlations with CA, validating their wettability alteration roles. Conversely, temperature, salinity, and NPs size display weak linear correlations (∣r∣ < 0.3) with CA, suggesting their influences operate via complex nonlinear mechanisms missed by Pearson diagnostics. These findings underscore the necessity of advanced nonlinear ML approaches (ANNs, XGBoost) to capture high-dimensional, non-monotonic interdependencies in nano-cEOR systems^38^.

### Machine learning model architectures

Six ML models (LR, RF, GBR, XGBR, ANN, ANN-RF) spanning simple linear to complex hybrid ensembles were developed for CA prediction:

1. Linear Regression (LR): Statistical baseline quantifying dataset non-linearity^26^.
2. Random Forest (RF): Ensemble algorithm constructing multiple decision trees; effective for tabular data, resistant to outliers, commonly used in EOR applications^4^.
3. Gradient Boosting (GBR): Sequential ensemble method where each tree corrects predecessor residuals via gradient descent, enhancing accuracy by focusing on larger prediction errors^72^.
4. Extreme Gradient Boost (XGBR): Highly optimized, regularized gradient boosting implementation with state-of-the-art performance on tabular data^38^.
5. Artificial Neural Network (ANN): Multi-layer perceptron (MLP) with four hidden layers (64–32-16–8 neurons) and tanh activation, designed to capture deep, complex non-linear relationships^27^.
6. ANN-RF Hybrid: Novel stacked ensemble combining optimized ANN and RF predictions as meta-features fed into a final LR model, leveraging ANN’s non-linear modeling with RF’s robust decision-making.

**Fig. 3:**
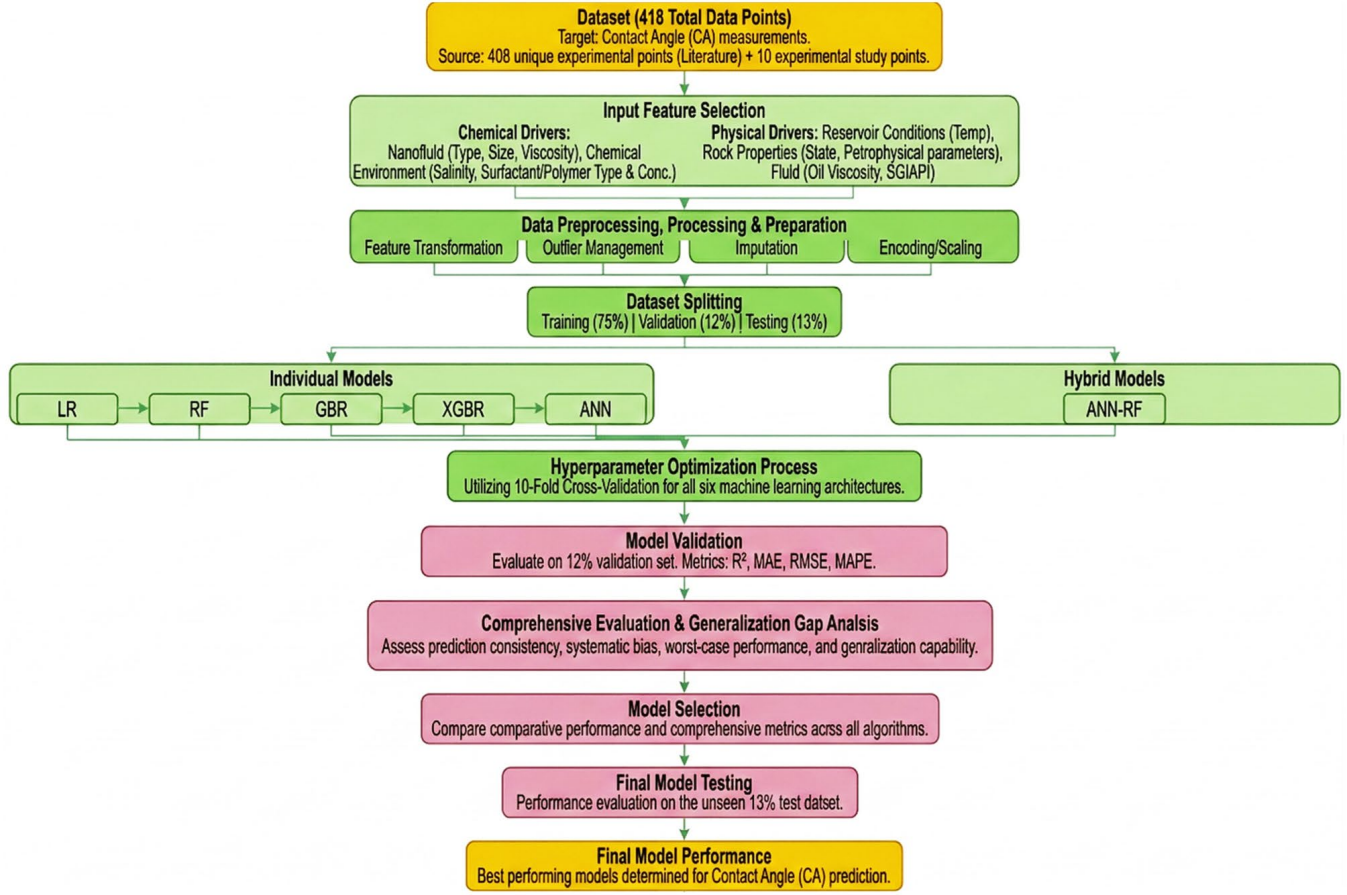
End-to-end ML framework schematic for contact angle prediction in nano-assisted chemical EOR. The workflow encompasses five integrated stages: (1) Data Input; (2) Preprocessing Pipeline; (3) Model Development; (4) Performance Evaluation; and (5) Sensitivity Analysis and Deployment.

Figure 3 presents schematic end-to-end ML workflow developed for CA prediction and model optimization.

### Hyperparameter optimization process

Hyperparameter optimization was performed via systematic grid search for RF, XGBR, GBR, LR, and ANN to maximize predictive accuracy and generalization. Detailed search ranges are provided in Supplementary Material S2 (Table 1). RF optimization yielded optimal performance with 100 estimators, max_depth of 20, log2 feature selection, no bootstrap, and minimal splitting parameters (MSE = -0.0101). XGBR achieved lowest MSE (-0.0094) at 150 estimators, max_depth of 13, learning_rate of 0.3, subsample and colsample_bytree at 0.8, gamma at 0, reg_alpha at 0.1, and reg_lambda at 2.0. GBR optimized to 300 estimators, learning_rate of 0.05, max_depth of 25, subsample of 0.7, with min_samples_split of 5 and min_samples_leaf of 2 (MSE = -0.0099). ANN architecture comprised four hidden layers (265 neurons each) with tanh activation, compiled using MSE loss and Adam optimizer (learning_rate = 0.001). LR optimization explored regularization methods (Ridge, Lasso, ElasticNet), with Ridge (alpha = 1.0, auto solver) demonstrating superior multicollinearity handling and overfitting prevention.

### Model evaluation metrics

Model performance was quantified using four standard statistical metrics: R^2^ (coefficient of determination), MAE (mean absolute error), RMSE (root mean squared error), and MAPE (mean absolute percentage error), which is particularly valuable for assessing relative variations across datasets with different scales, such as EOR applications where reservoir parameters exhibit high variability^73^. All six ML algorithms were further evaluated using comprehensive performance metrics including prediction consistency (residual standard deviation), systematic bias (mean residual), worst-case performance (min/max residuals), and generalization capability (training-validation gap). Detailed mathematical formulations are provided in Supplementary Material S1.

## Results and discussion

### Results

#### Comparative performance of ML models

The primary objective was to identify the most accurate and robust model for CA prediction. All six models were trained on the 334-point training set and evaluated on the 84-point unseen test set (testing and validation). The performance metrics are summarized in Table 3.

**Table 3:** Comparative performance metrics of Six ML models on training, testing, and validation datasets.

Model	Dataset	RMSE (°)	MAE (°)	MAPE (%)	R^2^ Score
LR	Training	24.99	20.15	44.39	0.68
	Testing	21.81	16.81	33.79	0.77
	Validation	21.99	17.65	34.71	0.73
RF	Training	8.41	5.76	11.58	0.96
	Testing	13.39	9.88	19.93	0.91
	Validation	13.08	9.17	19.45	0.90
GBR	Training	3.31	1.05	4.46	0.99
	Testing	11.85	8.87	17.58	0.93
	Validation	11.50	8.61	17.94	0.93
XGBR	Training	3.87	1.27	4.92	0.99
	Testing	11.61	9.05	17.36	0.93
	Validation	9.24	5.87	13.60	0.95
ANN	Training	7.59	5.62	12.54	0.97
	Testing	14.65	10.54	23.43	0.89
	Validation	13.76	10.20	18.94	0.89
ANN-RF	Training	6.06	4.10	9.13	0.98
	Testing	10.18	7.03	16.45	0.95
	Validation	12.60	8.90	17.70	0.91

**Fig. 4:**
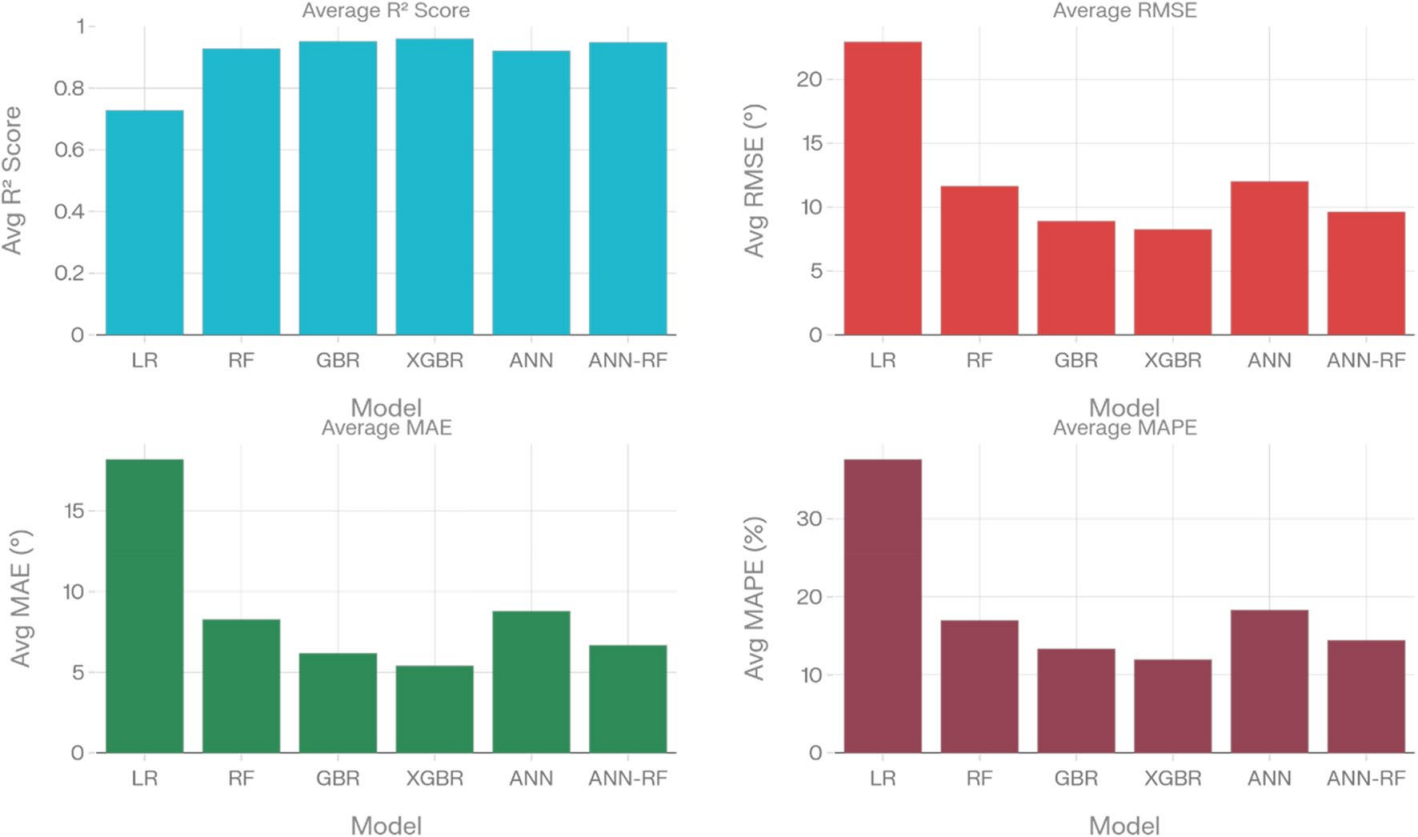
Average performance metrics (R^2^, RMSE, MAE, MAPE) across all datasets for each ML model.

The results clearly indicate the superior performance of ensemble and hybrid models over traditional linear approaches for CA prediction. The baseline LR model performed poorly, with an average R^2^ of 0.73 (Fig. 4), confirming the highly non-linear nature of the wettability problem. In contrast, tree-based ensembles (RF, GBR, XGBR), ANN, and the hybrid ANN-RF demonstrated substantially higher average R^2^ values of 0.93, 0.95, 0.96, 0.92, and 0.95, respectively. Figure 4 presents average performance metrics (R^2^, RMSE, MAE, MAPE) across all datasets. XGBR achieved the highest validation accuracy (Table 3) demonstrating strong ability to capture complex wettability mechanisms while maintaining excellent generalization. The ANN-RF Hybrid ranked second, effectively combining neural network pattern recognition with Random Forest ensemble robustness. GBR achieved comparable metrics to XGBR.

While averaged metrics provide consolidated rankings, the detailed breakdown in Table 3 reveals critical generalization characteristics. GBR and ANN exhibited overfitting, with training R^2^ values of 0.99 and 0.97 degrading to testing scores of 0.93 and 0.89, respectively. XGBR demonstrated best generalization control, attributed to its built-in regularization mechanisms. The ANN-RF Hybrid similarly maintained strong generalization, validating the stacked ensemble architecture’s effectiveness in mitigating overfitting.

**Fig. 5:**
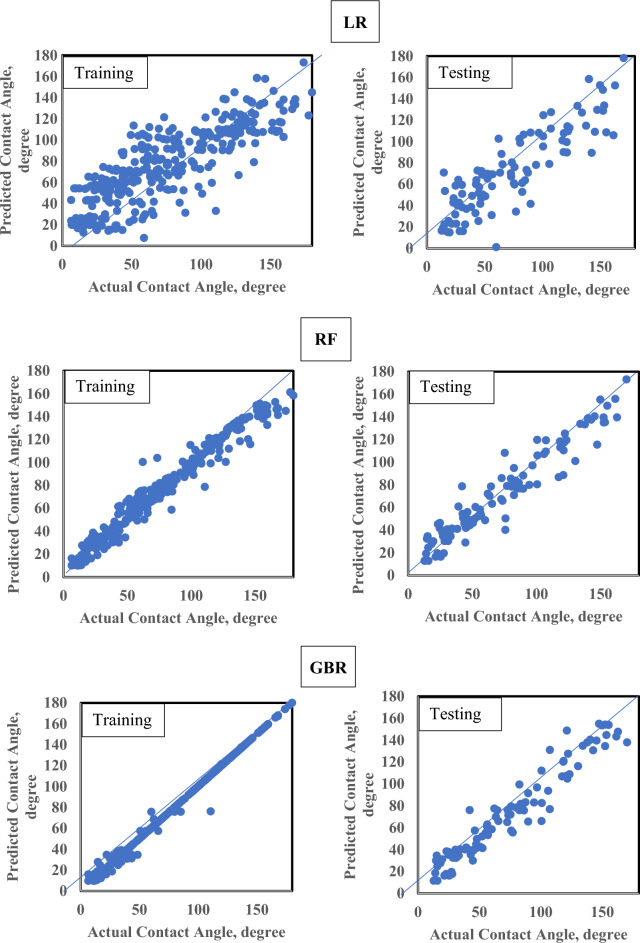

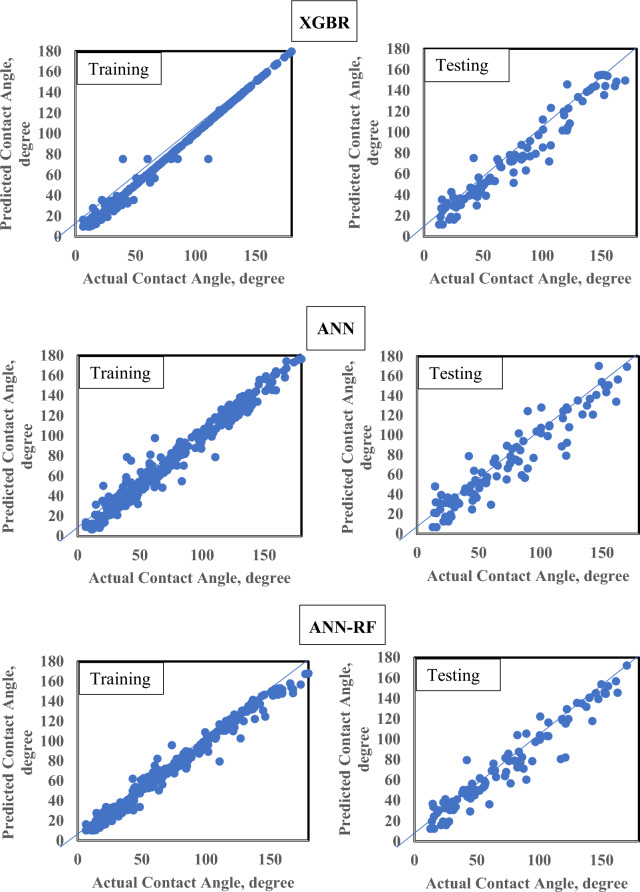
Actual versus predicted CA values for training and testing datasets across all evaluated models, showing regression fit and prediction accuracy.

**Fig. 6:**
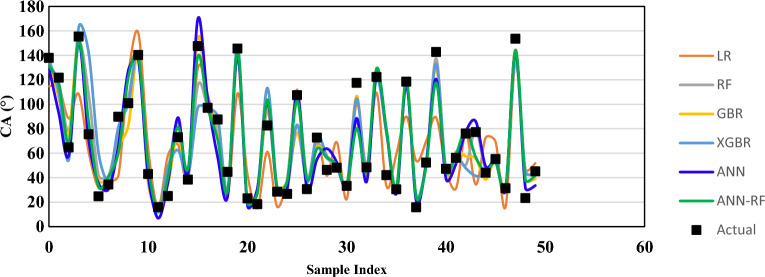
Actual (black dots) versus predicted (continuous lines) CA values for the validation dataset across all six models, demonstrating model generalization performance and prediction reliability on unseen data.

Visual analytics through "Actual vs Predicted" plots further corroborate these findings. Figure 5 presents the training and testing dataset predictions, while Fig. 6 shows validation dataset performance. XGBR and ANN-RF exhibit tighter clustering around the ideal fit line and more consistent prediction distributions across all splits, reflecting their superior metrics and robustness. The moderate-performing ANN and RF models remain viable alternatives but require additional regularization strategies to close generalization gaps. Overall, the results confirm that advanced ensemble and hybrid architectures provide the most reliable and accurate predictions for CA prediction in nano-cEOR applications.

#### Model generalization and residual diagnostics

**Fig. 7:**
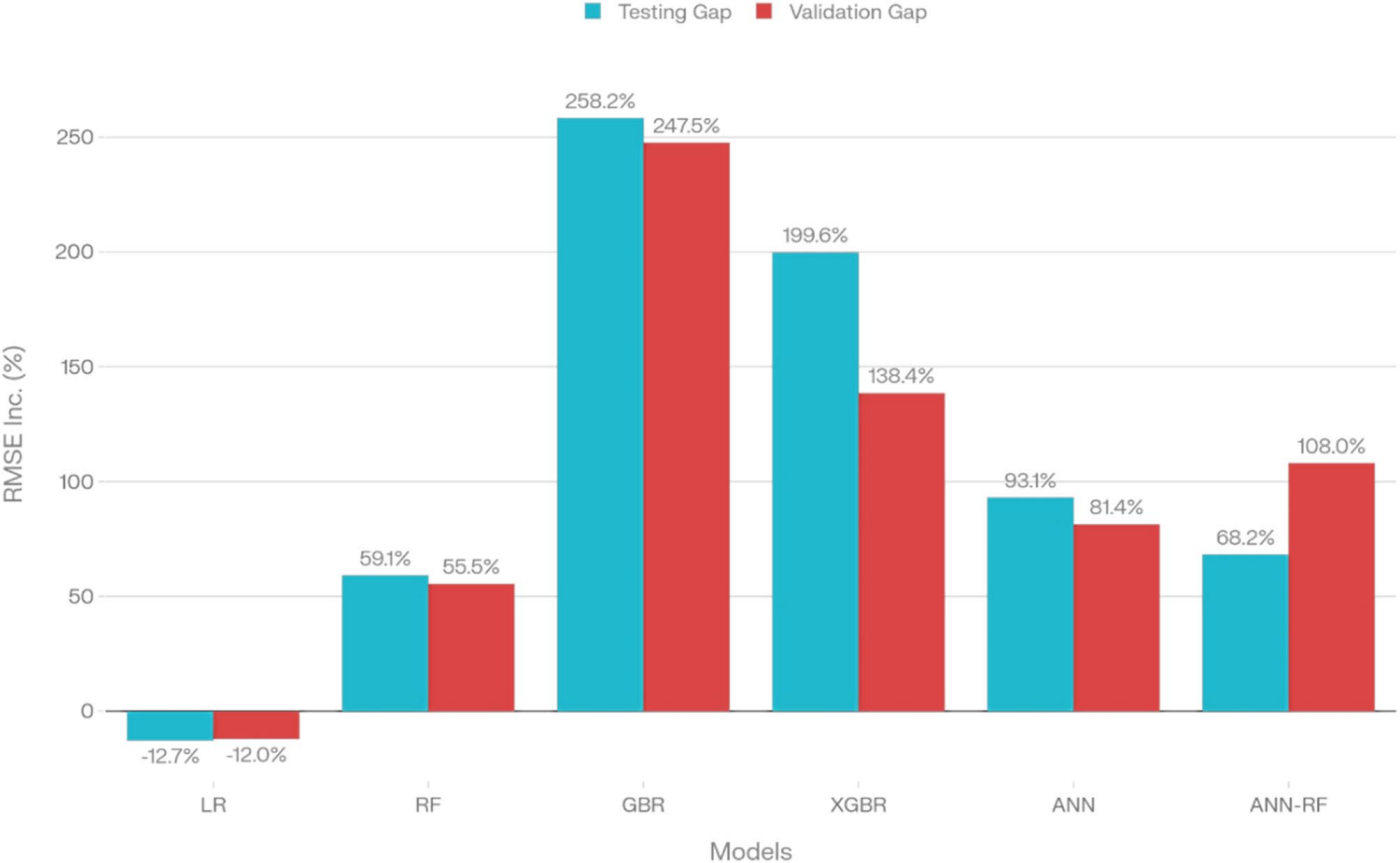
Generalization gap analysis for the six developed ML models.

Six ML models (LR, RF, GBR, XGBR, ANN, ANN-RF) were evaluated for CA prediction using training, testing, and validation splits. Performance was assessed via RMSE, MAE, MAPE, R^2^, residual statistics (standard deviation, mean, min/max), and generalization gap **(**Table 4 and Fig. 7).

**Table 4:** Validation performance metrics.

Model	Residual std Dev (°)	Mean residual (°)	Min residual (°)	Max residual (°)	Generalization gap (%)
LR	21.79	2.94	-37.89	53.37	-11.98
RF	13.03	-1.12	-36.63	32.04	55.45
GBR	11.45	1.02	-33.99	30.49	247.51
XGBR	9.24	-0.04	-33.13	24.53	138.39
ANN	13.73	1.03	-36.60	30.94	81.38
ANN-RF	12.59	-0.39	-37.31	37.02	108.04

XGBR demonstrated superior validation performance (R^2^ = 0.95, RMSE = 9.24°, MAE = 5.87°, MAPE = 13.60%) with the smallest residual standard deviation (9.24°), near-zero mean residual (-0.04°), and controlled residual range (-33.13° to + 24.53°), indicating minimal systematic bias. Its training-to-validation RMSE increase of 138% substantially outperformed GBR’s excessive variance (248% increase). The ANN-RF hybrid exhibited the strongest generalization among ensemble models (108% increase, residual std = 12.59°, mean = -0.39°), suggesting optimal bias-variance tradeoff. RF showed moderate generalization (55% increase, residual std = 13.03°), while ANN exhibited intermediate overfitting (81% increase, residual std = 13.73°). In contrast, LR underperformed across all metrics (R^2^ = 0.73, RMSE = 21.99°, residual std = 21.79°) with a negative generalization gap (-11.98%), confirming underfitting and fundamental inadequacy for capturing nonlinear dependencies.

**Fig. 8:**
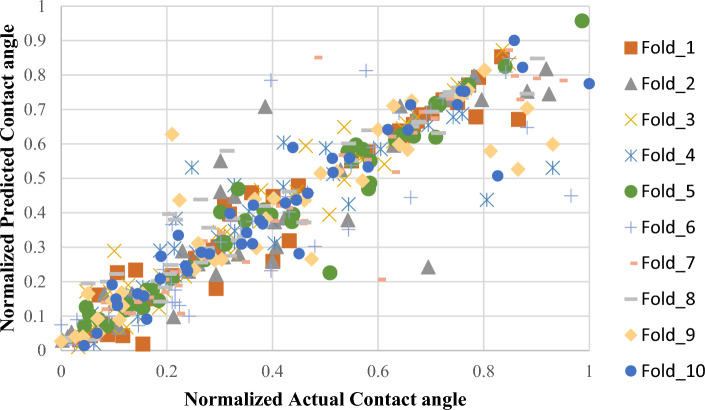
Normalized predicted versus normalized actual CA values CV performance of XGBR model across tenfold partitioning.

**Fig. 9:**
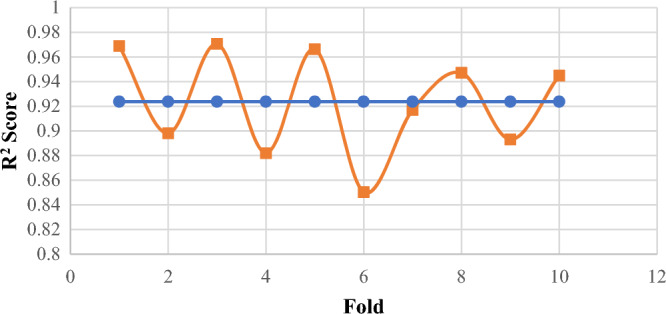
XGBR model CV performance of across tenfold partitioning. R^2^ scores for each fold (orange line) compared to the overall average R^2^ (blue line).

To validate model stability, tenfold cross-validation was implemented on XGBR. The normalized predicted-versus-actual scatter plot (Fig. 8) demonstrated strong linear correlation across the entire CA range (6.5°–180°) with minimal deviation. The fold-averaged R^2^ of 0.924 ± 0.067 (range: 0.723–0.943) closely matched independent validation performance (R^2^ = 0.953), confirming robust generalization without data-specific bias (Fig. 9). Notably, 8 of 10 folds achieved R^2^ > 0.85, with moderate degradation in Folds 4 (0.778) and 6 (0.723) likely attributable to challenging samples concentrated in those partitions.

#### Comprehensive sensitivity analysis

Global and local sensitivity analyses were conducted to elucidate parameter importance hierarchies, quantify non-linear response behaviors, and identify synergistic interactions governing CA predictions. The sensitivity analysis was performed by generating 5,000 synthetic input samples spanning the full dataset range via Monte-Carlo sampling: continuous variables were sampled uniformly between observed minimum and maximum values, while categorical variables (Sample State, Rock Type, NPs type, Chemical Additive) were assigned integer codes and sampled discretely across all categories. For each realization, the trained XGBoost model predicted the resulting CA, producing a high-resolution synthetic dataset linking randomized inputs to model outputs for robust parameter influence estimation. This multi-method framework integrates variance-based Sobol indices for continuous variables, SHAP values for unified feature attribution, Pearson/Spearman correlations for monotonicity assessment, partial dependence plots (PDPs) for response characterization, and cumulative distribution functions (CDFs) for distributional validation^74,75^. The complementary application of these methods addresses inherent limitations of individual techniques while providing robust cross-validation of sensitivity rankings^76^.

##### Global feature importance

**Fig. 10:**
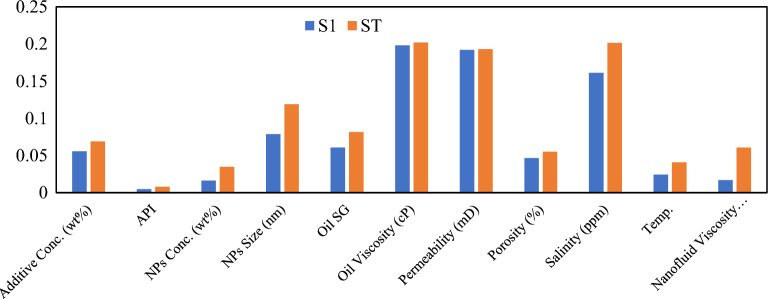
Sobol analysis first and total order effect.

Variance-based Sobol sensitivity analysis decomposes total predictive variance into first-order effects (S1) and total-order effects (ST) for continuous reservoir and fluid properties **(**Fig. 10**)**. First-order indices reveal a hierarchical structure dominated by oil viscosity (S1 = 0.198, 95% CI “Confidence Interval”: 0.184–0.212), permeability (S1 = 0.192, CI: 0.179–0.205), and salinity (S1 = 0.162, CI: 0.149–0.175), collectively explaining 55.2% of CA variance. Secondary parameters include NPs size (S1 = 0.078), Oil SG (S1 = 0.060), and Porosity (S1 = 0.045).

The divergence between first-order and total-order indices (ST − S1) quantifies interaction strength. Salinity exhibits the largest interaction component (ST − S1 = 0.040), indicating substantial synergistic effects. Notably, NPs concentration (S1 = 0.005, ST = 0.025) and chemical additive concentration (S1 = 0.05, ST = 0.067) display disproportionately high total-order effects, with chemical additive concentration exhibiting the highest interaction ratio (ST/S1 = 13.4), signifying near-complete dependence on co-variation with nanoparticle loading; consistent with polymer-NPs dispersion stabilization mechanisms^77^.

**Fig. 11:**
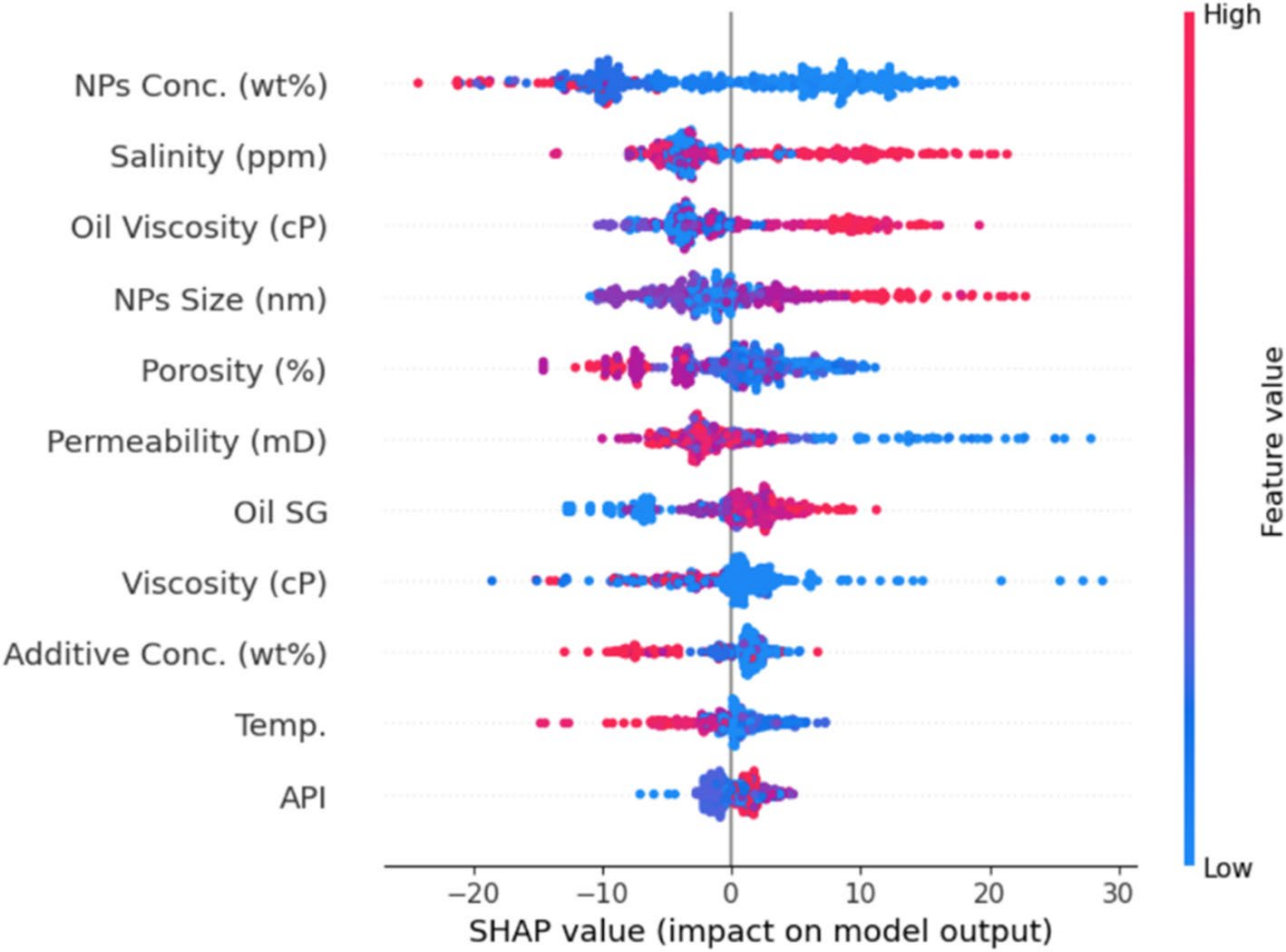
SHAP feature importance analysis and ranking.

SHAP feature importance analysis confirms sensitivity quantification to continuous **(**Fig. 11**)**. SHAP formulation parameters exert substantial influence: NPs size (7.6°) and porosity (5.9°). Aggregation was performed by summing SHAP values across one-hot encoded dummy variables to ensure proper group-level attribution^74^.

CV between Sobol and SHAP rankings demonstrates strong concordance for continuous variables: oil viscosity (Sobol rank 1, SHAP rank 3), permeability (Sobol rank 2, SHAP rank 2), and salinity (Sobol rank 3, SHAP rank 4). Minor discrepancies arise from methodological differences; Sobol quantifies global variance contribution while SHAP measures average marginal prediction shifts^75^. Salinity ranks higher in SHAP (mean |SHAP|= 9.4°) than Sobol variance contribution suggests, reflecting sharp response gradients at extreme salinity values (> 100,000 ppm).

The most pronounced ranking divergence occurs for NPs concentration: negligible Sobol S1 = 0.005, rank 10; but top-tier SHAP contribution (mean |SHAP|= 6.8°, rank 5). This is reconciled by dataset distribution—82% of samples contain 0.05–0.30 wt% NPs (narrow range producing minimal Sobol variance), while the remaining 18% span 0.30–1.50 wt% exhibiting strongly non-linear responses (CA reductions exceeding 20°). SHAP’s instance-level attribution captures these extreme marginal effects while Sobol’s global variance averaging dilutes their contribution^78^.

**Fig. 12:**
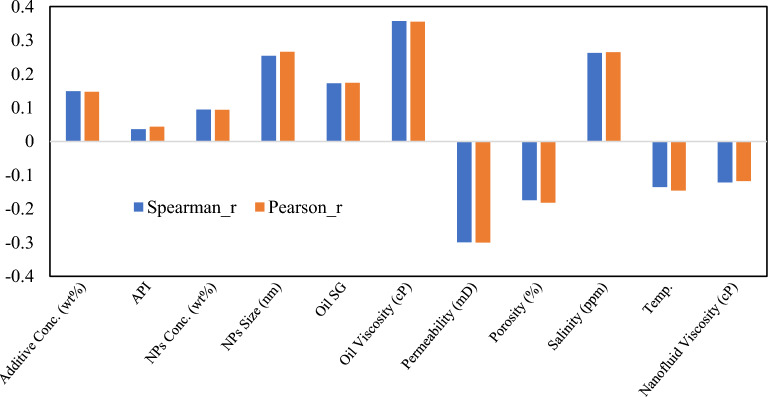
Spearman and Pearson correlation coefficients.

Pearson and Spearman correlation coefficients provide pairwise relationship assessments **(**Fig. 12**)**. Near-perfect concordance (mean absolute difference = 0.008) indicates predominantly linear relationships. Oil viscosity exhibits the strongest positive correlation (r = 0.36, p < 0.001), permeability strong negative correlation (r =  − 0.30, p < 0.001), and salinity positive correlation (r = 0.26, p < 0.001), though subsequent analysis reveals this relationship is non-monotonic with an optimal window rather than linear trend. NPs concentration exhibits weak correlation (r = 0.09, p = 0.002) despite moderate SHAP importance, indicating threshold-dependent non-linearity.

##### Continuous parameter response curves

**Fig. 13:**
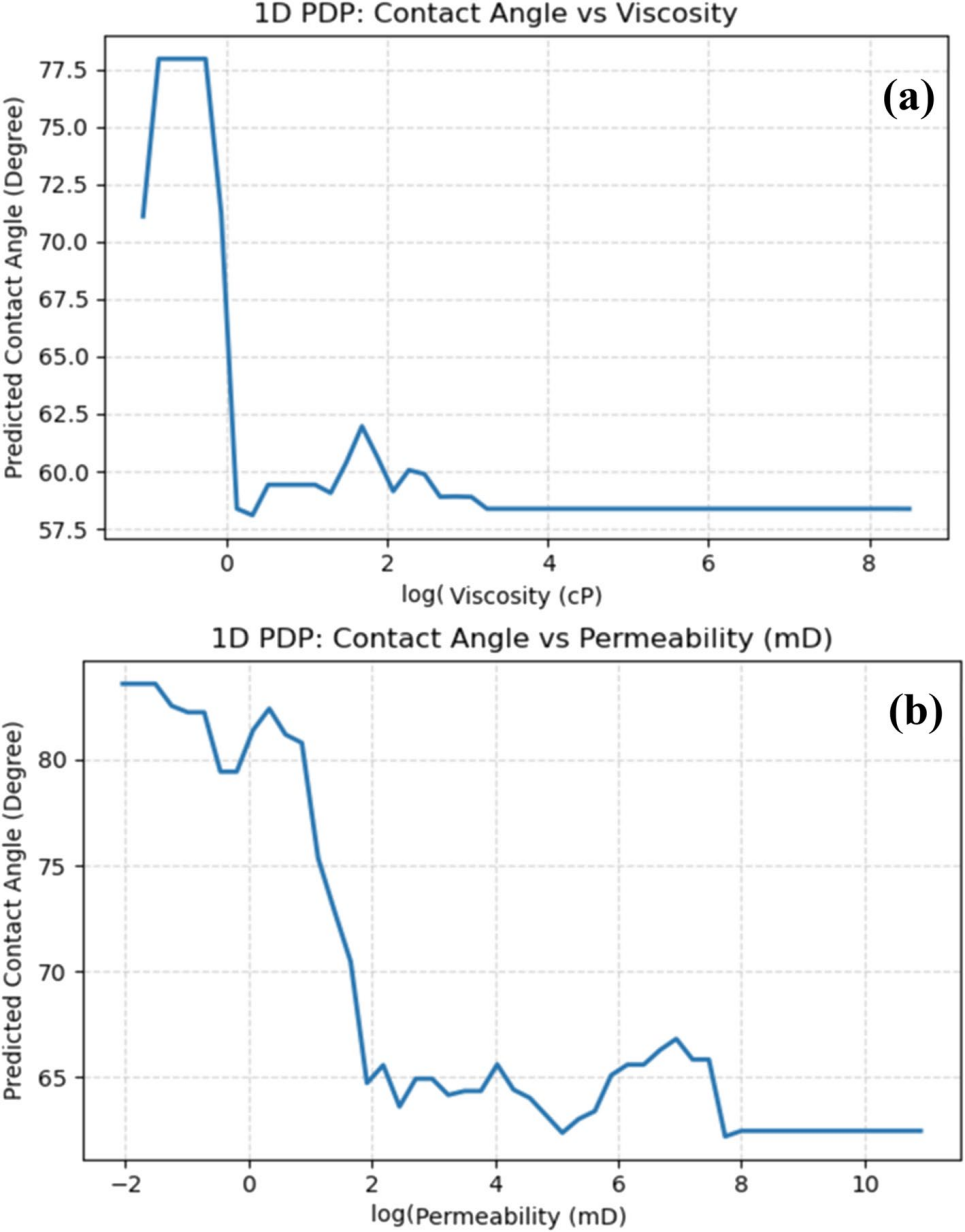
1D PDP of Ppredicted CA versus (**a**) viscosity, and (**b**) permeability.

**Fig. 14:**
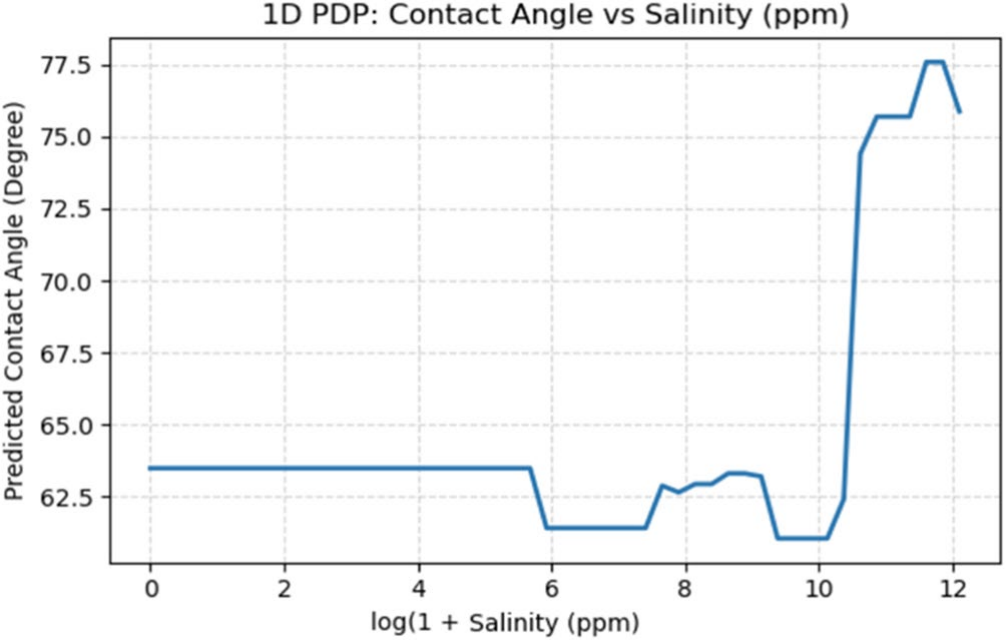
1D PDP of predicted CA vs salinity (ppm).

Partial dependence plots for key continuous parameters reveal non-linear responses and critical threshold effects (Figs. 13 and 14). Nanofluid viscosity (Fig. 13a) exhibits sharp non-monotonic behavior: CA peaks at near-zero viscosity (dry conditions), declines rapidly as viscosity increases to ~ 0.5 cP (aqueous phase establishment), then plateaus beyond 1.5 cP. This transition at 0.2–0.5 cP marks the critical threshold for NPs suspension stability, where increasing aqueous viscosity enhances NPs dispersion by counteracting aggregation through elevated drag forces^79^.

Permeability demonstrates dramatic threshold-driven behavior with three distinct regimes (Fig. 13b): CA remains elevated in ultra-tight rocks (K < 0.1 mD) where NPs cannot penetrate pore networks, undergoes steep decline across the critical transition zone (0.1–3 mD), then stabilizes in permeable formations (K ≥ 3 mD) where surface chemistry governs wettability. The 0.1 mD threshold indicates nano-cEOR is ineffective in tight reservoirs without hydraulic fracturing^76^.

Salinity exhibits complex U-shaped response (Fig. 14): CA remains relatively stable at low salinity (0–10,000 ppm), decreases moderately in the optimal window (30,000–80,000 ppm) corresponding to effective ion bridging between negatively charged NPs and carbonate surfaces, then increases sharply at hypersaline conditions (> 100,000 ppm) due to aggregation-inducing ionic compression^77^. This trend validates that moderate-salinity nanofluids outperform both low- and hypersaline formulations.

##### Categorical parameter distributional analysis

**Fig. 15:**
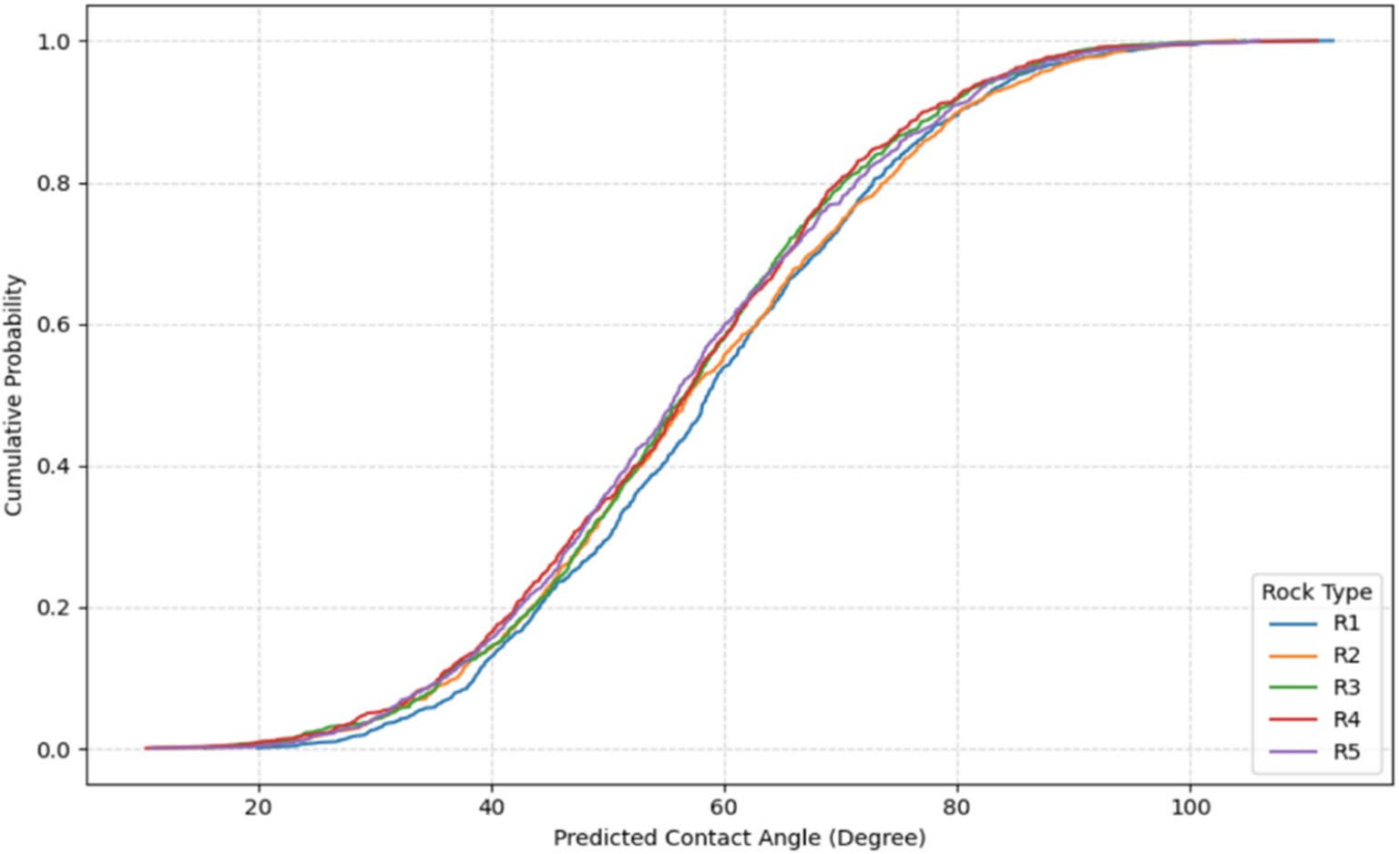
CDF of CA at different rock types.

**Fig. 16:**
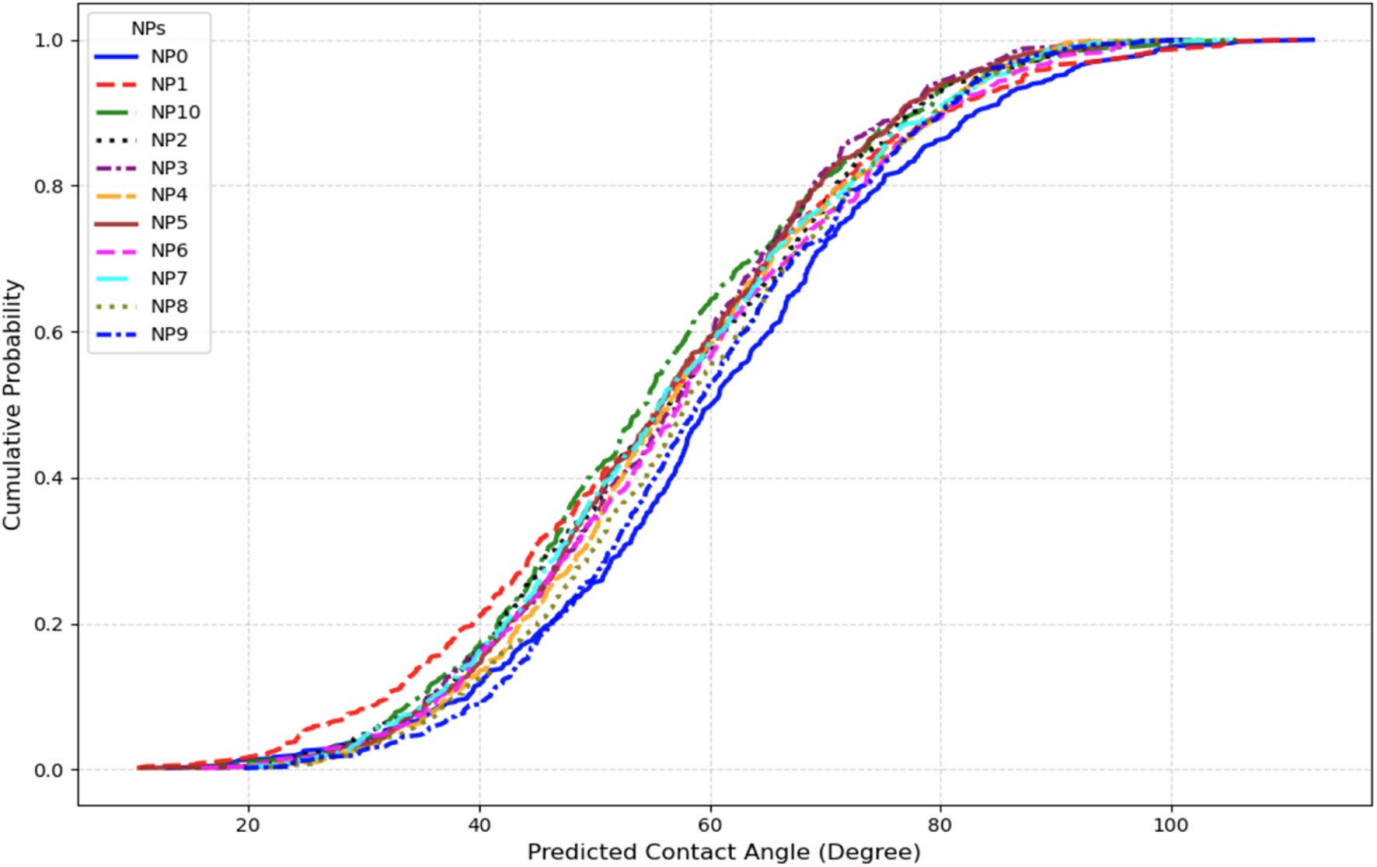
CDF of CA at different NPs types.

**Fig. 17:**
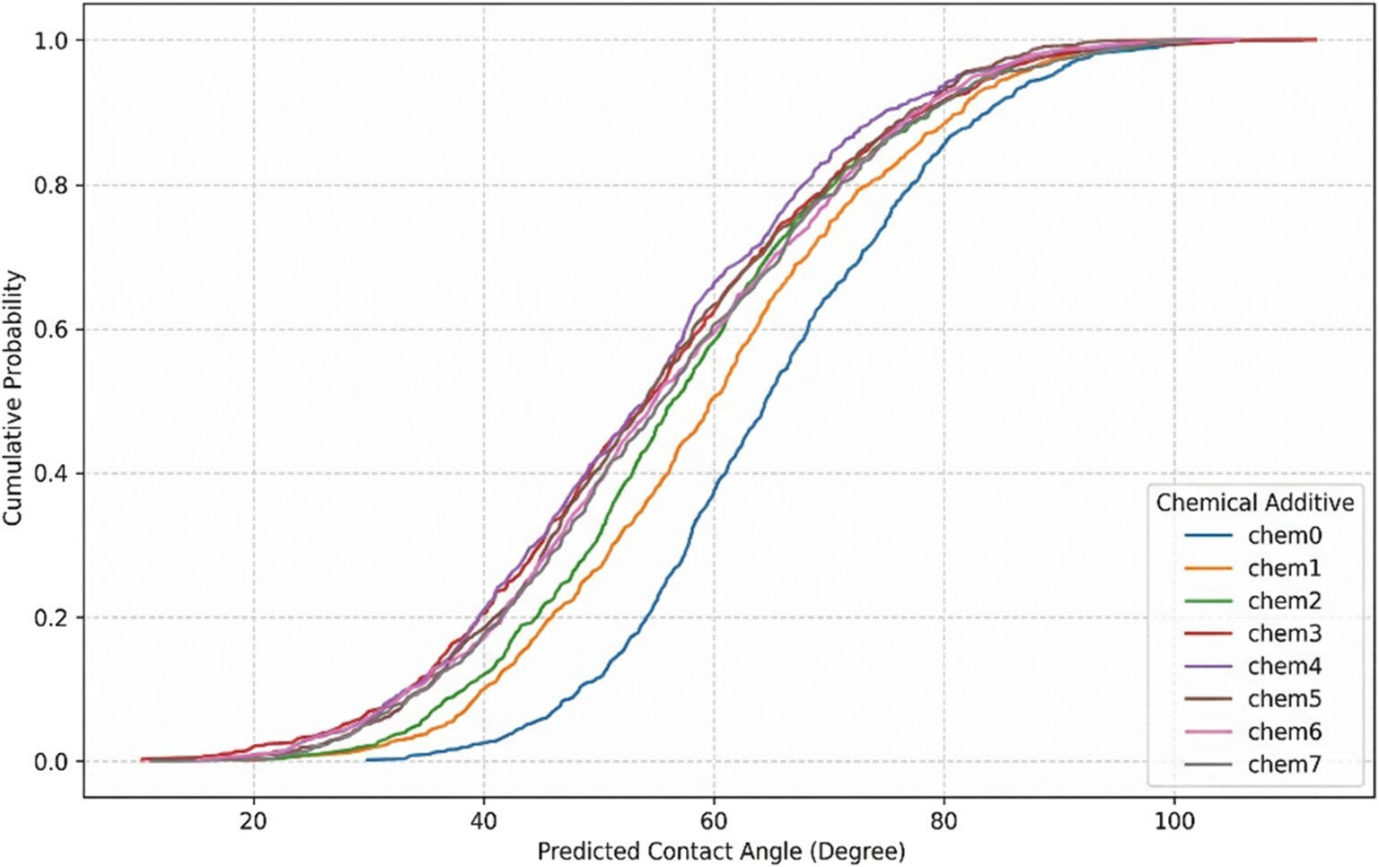
CDF of CA at different chemical additives.

**Fig. 18:**
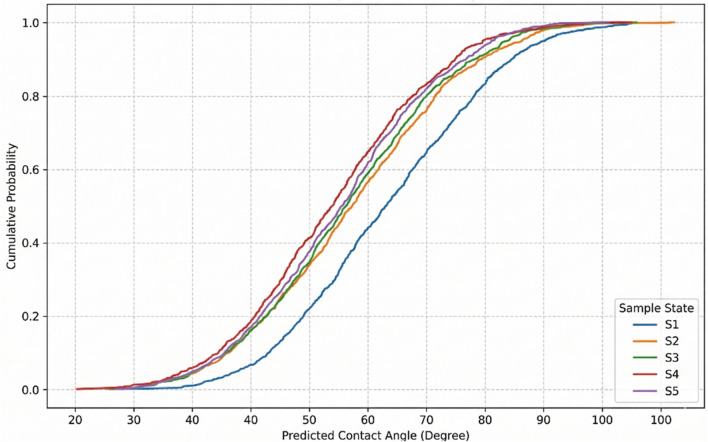
CDF of CA at different sample states.

Cumulative distribution function stratification quantifies full distributional characteristics and performance variability across categorical variables (Figs. 15, 16, 17, 18). Rock type CDFs (Fig. 15) reveal significant lithology-dependent wettability: dolomite (R4) exhibits the most oil-wet distribution, followed by limestone (R1), with near-complete overlap between limestone and carbonate (R5) confirming identical lithologies. Sandstone (R2) displays intermediate wettability, while glass (R3) shows the narrowest distribution, reflecting substrate uniformity^78^. NPs type CDFs (Fig. 16) establish clear performance hierarchy. ZrO_2_ (NP6), Fe_3_O_4_ (NP4), CuO (NP7), and NiO (NP5) demonstrate superior wettability alteration with leftward-shifted distributions, attributed to high zeta potential (> 25 mV) and small hydrodynamic diameters (10–30 nm). SiO_2_ (NP1) exhibits weakest effectiveness despite extensive literature coverage. Progressive distribution narrowing from control (NP0) to high-performance NPs indicates that effective NPs reduce performance variability, enhancing field deployment predictability^79^.

Chemical additive CDFs (Fig. 17) demonstrate biosurfactant (chem4) superiority with the lowest median CA and narrowest distribution, achieving substantially greater wettability alteration than anionic surfactants (chem1) due to amphoteric charge adaptation on mineral surfaces. Polymer formulations (chem6, chem7) exhibit performance comparable to nonionic surfactants, representing synergistic polymer-NPs interactions consistent with negligible Sobol S1 (0.005) but substantial ST (0.067) for additive concentration^77^.

Sample state CDFs (Fig. 18) reveal aging protocol effects: oil-aged samples (S1) exhibit the most oil-wet distribution, while high-salinity nanofluid-aged samples (S3) show modest improvement over low-salinity (S4), validating enhanced wettability alteration via divalent cation-mediated ion bridging at elevated salinity (> 30,000 ppm), though substantial CDF overlap indicates context-dependent advantage^76^.

##### Synergistic interaction mapping

**Fig. 19:**
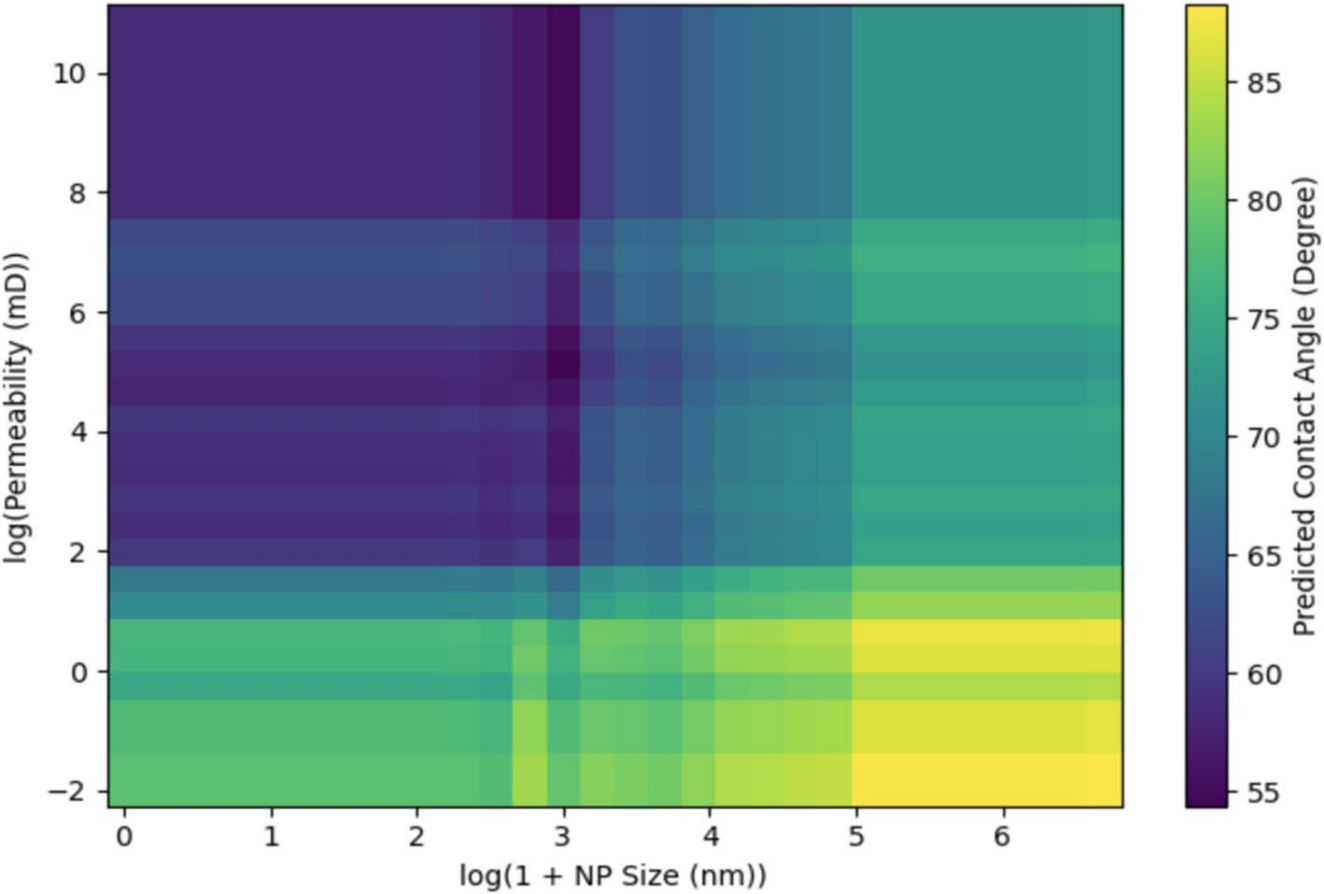
2D PDP quantify synergistic interaction between NPs size and permeability and its effect on CA.

**Fig. 20:**
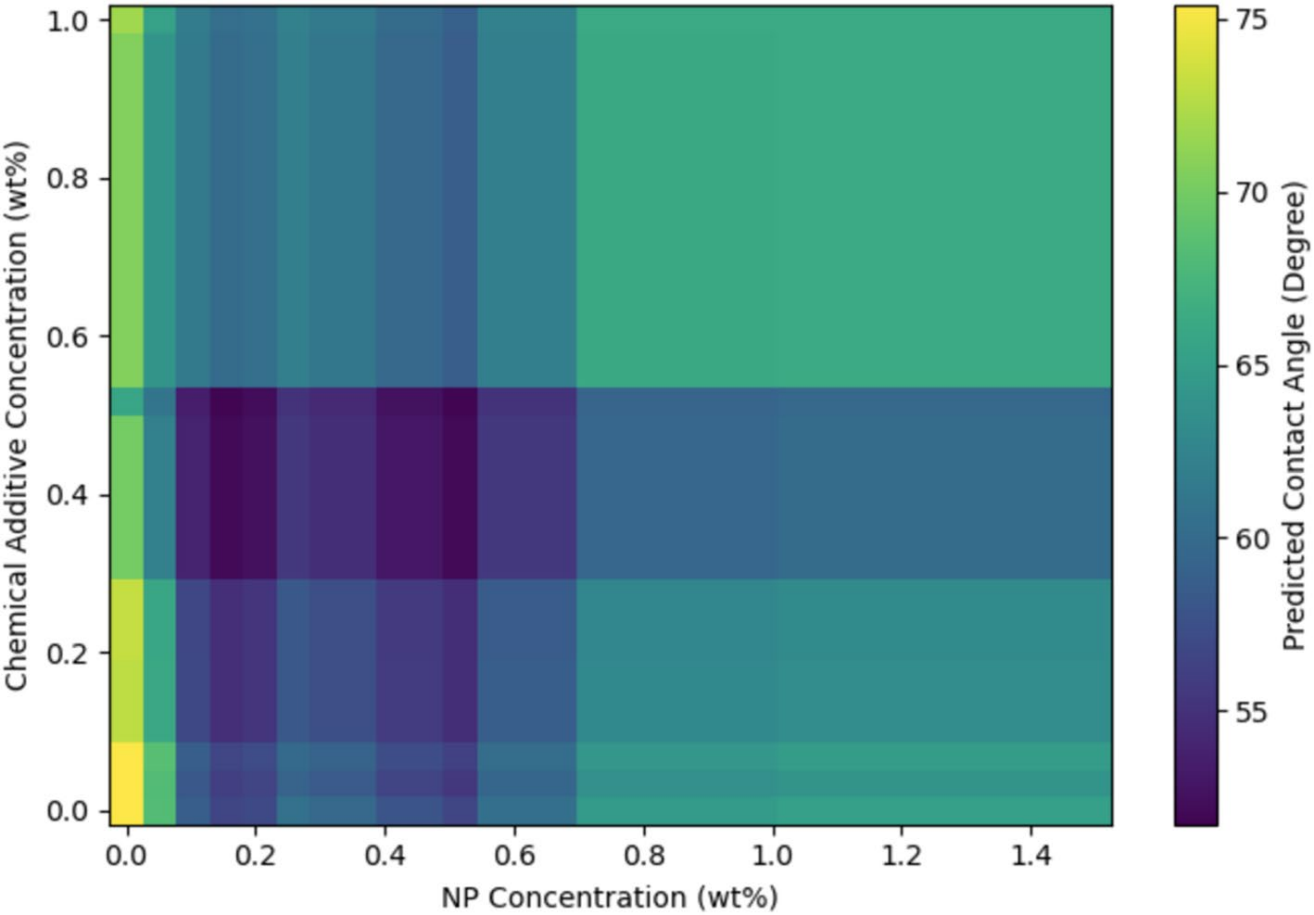
2D PDP quantify synergistic interaction between NPs concentration and chemical additive concentration and its effect on CA.

Two-dimensional PDP quantify synergistic interactions revealing optimal operational windows (Figs. 19 and 20). NPs size – permeability interaction (Fig. 19) reveals critical size-accessibility coupling. Optimal performance zone (CA 55–60°) corresponds to small NPs (10–30 nm) in moderate-to-high permeability rocks (1 – 1,000 mD). Poorest performance (85–90°) occurs where small NPs encounter ultra-tight rocks (K < 0.1 mD): transport is prohibited despite favorable surface chemistry^75^. Large NPs (> 50 nm) exhibit uniformly degraded performance across all permeabilities (70–80°). Sharp transition at K ≈ 0.1–3 mD delineates accessibility threshold below which size optimization becomes irrelevant. This establishes permeability-dependent sizing guideline: tight carbonates (K = 0.5–5 mD) require 10–20 nm formulations, conventional sandstones (K = 50–500 mD) accommodate 20–40 nm particles, high-permeability formations (K > 500 mD) can utilize 40–80 nm NPs^78^.

NPs concentration – chemical additive concentration interaction (Fig. 20) exposes synergistic zones. Three operational regimes emerge: (1) Sub-threshold loading (< 0.1 wt%) produces poor performance (70–76°) regardless of additive concentration; (2) Intermediate loading (0.1–0.5 wt%) yields moderate performance (58–65°) with minimal additive sensitivity; (3) High loading (0.5–1.0 wt%) exhibits strong additive dependence—low additive (< 0.3 wt%) results in degraded performance (60–68°) from aggregation, whereas high additive (> 0.5 wt%) achieves optimal performance (52–55°) through steric stabilization^77^. This synergistic zone (0.5–1.0 wt% NPs and 0.5–0.8 wt% additive) achieves 6–8° additional reduction compared to equivalent NPs concentrations with low additive, quantifying polymer–NPs synergy. Optimal concentration ratio for loadings exceeding 0.5 wt% is approximately 1 to 1 up to 1.5 to 1 (NPs to additive mass ratio). Upper boundary at 1.0 wt% NPs, beyond which performance degrades (65–75°), likely reflects pore clogging or viscosity increases impairing injectivity^79^.

##### Rock-type conditional sensitivity

**Fig. 21:**
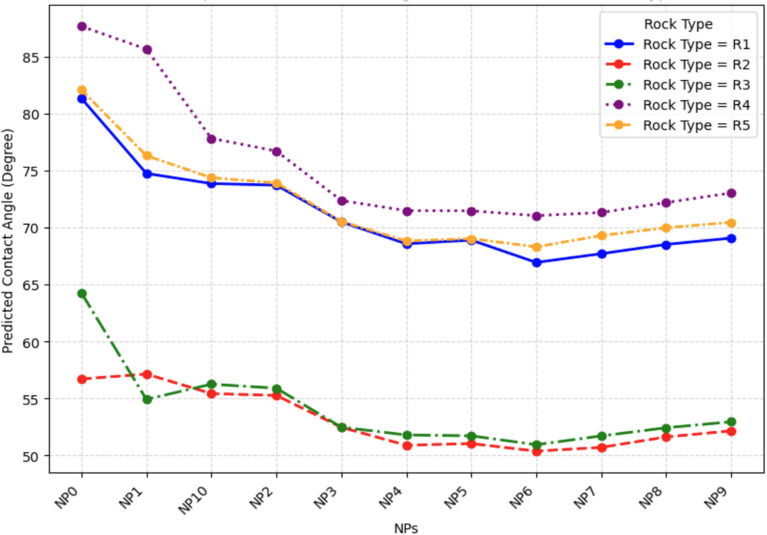
Partial dependence plot of CA on NPs for different rock types.

**Fig. 22:**
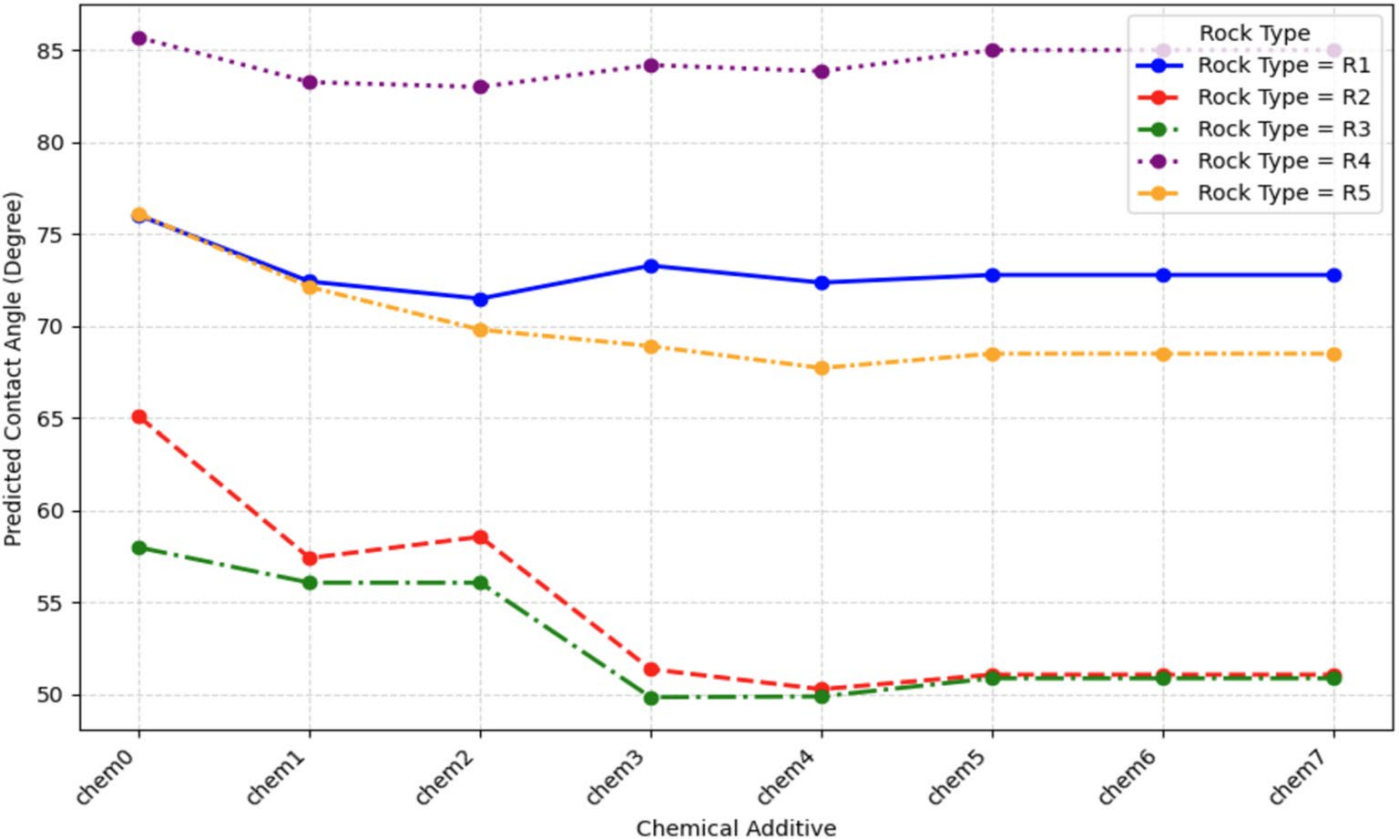
Partial dependence plot of CA on chemical ddditives for different rock types.

Conditional partial dependence analysis stratified by rock type reveals lithology-dependent effectiveness enabling reservoir-specific optimization (Figs. 21 and 22). NPs performance rankings vary substantially across rock types (Fig. 21). On limestone (R1, baseline median = 81°), ZrO_2_ achieves maximum reduction (21°, final = 60°), followed by TiO₂ (18°, final = 63°), Fe_3_O_4_ (16°, final = 65°), and Al₂O₃ (13°, final = 68°). Large reduction magnitude reflects highly oil-wet baseline and reactive calcite surface chemistry facilitating strong electrostatic interactions^76^.

Sandstone (R2, baseline = 70°) exhibits compressed differentiation: ZrO_2_ (14°, final = 56°), Fe₃O₄ (13°, final = 57°), CuO (12°, final = 58°), and TiO_2_ (11°, final = 59°). Narrower reduction range indicates negatively charged silica imposes different adsorption energetics than positively charged carbonate. Dolomite (R4, baseline = 87°) achieves only moderate maximum reduction (16° for ZrO_2_, final = 71°), suggesting mixed Ca-Mg surface chemistry creates additional barriers. Notably, MgO exhibits enhanced relative performance on dolomite (14° reduction, ranking 4th) compared to overall rankings (8th), indicating statistically enhanced model-predicted performance on dolomite surfaces that is mechanistically consistent with the higher affinity of MgO NPs for Mg^2+^-enriched mineral surface sites documented in adsorption isotherm studies^80^. It is emphasized that this interpretation reflects a statistical correlation from a limited number of dolomite samples within the compiled dataset,direct experimental confirmation of preferential Mg^2+^ site adsorption under nano-cEOR conditions is beyond the scope of this study and represents an important direction for future laboratory investigation.

ZrO_2_ emerges as universal top performer across all lithologies, establishing it as preferred choice for exploration applications where reservoir mineralogy is incompletely characterized. SiO₂ consistently underperforms (3–8° reductions), likely due to low zeta potential (< 15 mV) limiting electrostatic adsorption^77^.

Chemical additive effectiveness exhibits strong lithology dependence (Fig. 22). On carbonate substrates (positively charged surfaces), biosurfactants achieve maximum reductions (18° on limestone, 15° on dolomite), followed by anionic surfactants (12°) and anionic polymers (11°), reflecting favorable electrostatic attraction between negatively charged head groups and mineral surfaces. Cationic surfactants perform poorly (7–8°) due to charge repulsion. Biosurfactant superiority stems from amphoteric character, enabling adaptive charge configuration optimizing both rock adsorption and oil–water interfacial activity^79^.

On sandstone (R2, negatively charged silica), optimal hierarchy inverts: cationic surfactants emerge as top performers (11°, final = 56°), followed by nonionic surfactants (10°, final = 57°) and biosurfactants (9°, final = 58°). Anionic surfactants achieve modest effectiveness (7°), confirming charge-dependent adsorption as dominant mechanism. Universally strong performance of nonionic surfactants (9–10° across all rock types) positions them as robust choices for mixed-lithology reservoirs^76^.

### Discussion

This study presents a comprehensive benchmarking of ML models for CA prediction in nano-cEOR, leveraging a rigorously curated dataset spanning peer-reviewed publications over 2013–2025. The performance of six distinct models LR, RF, GBR, XGBR, ANN, and a hybrid ANN-RF was evaluated using an extensive suite of metrics on independent training, testing, and, most critically, validation splits. This approach follows established ML validation protocols and provides comprehensive diagnostic transparency, and directly addresses a recognized gap in the literature: the absence of transparent, model-agnostic validation and error characterization for CA prediction in EOR-relevant systems.

#### Interpretation of model performance

The results reveal several key insights regarding model selection. Firstly, the XGBR model achieved the highest performance on the reserved validation partition, with R^2^ = 0.953, RMSE = 9.24°, MAE = 5.87°, MAPE = 13.60%, and near-zero residual bias (mean residual: − 0.04°). It is important to note, however, that model ranking is metric- and partition-dependent. On the independent testing set, the ANN-RF Hybrid achieved R^2^ = 0.95, marginally superior to XGBR’s testing R^2^ = 0.93, while XGBR outperformed ANN-RF on the validation partition (R^2^ = 0.953 vs. 0.912). This inconsistency reflects the inherent variability of small held-out subsets and the complementary strengths of the two architectures: ANN-RF demonstrates stronger generalization within the testing partition while XGBR demonstrates more consistent performance on the most sequestered validation data. Both models are therefore considered strong candidates for deployment, and their combined use as ensemble predictors is recommended where computational resources permit. The primary model selection criterion applied here performance on the reserved validation set, favors XGBR, but practitioners operating within similar dataset characteristics should consider evaluating both models. For practical field screening, over 95% of XGBR validation errors fall within ± 18.5°, permitting actionable confidence intervals for nanofluid formulation screening.

The GBR also performed well, with validation RMSEs of 12.60° and 11.50°, and R^2^ of 0.927. However, GBR exhibited a pronounced generalization gap (248%), indicating overfitting, whereas XGBR and ANN-RF showed more controlled, acceptable generalization gaps (138% and 108%, respectively). LR, by contrast, exhibited persistently poor predictive power, with validation R^2^ of only 0.731, MAPE of 34.7%, and the largest maximum residuals (–44.6° to + 46.6°), confirming its inadequacy for capturing the nonlinear relationships present in CA data^26^. This is an expected outcome, given the complex, threshold-based physical mechanisms (e.g., disjoining pressure, critical micelle concentration) at play. Figure 5 illustrate the comparative average validation performance of all datasets for all models across RMSE, MAE, MAPE, and R^2^ metrics, while Table 3 summarizes each model’s full analytic profile, including critical diagnostic parameters (residual bias, spread, extreme residuals, and generalization gap). Compared to state-of-the-art literature, which commonly reports CA prediction errors ranging from 12–25° RMSE with R^2^ values typically below 0.90 when assessed on holdout datasets without proper CV or external validation, the present results demonstrate a marked improvement in both accuracy and reliability.

#### Key mechanistic insights from ML-derived thresholds

The integrated sensitivity framework reveals operational thresholds and rock-specific optimization strategies that advance beyond established EOR physics into quantitative, experimentally testable guidelines.

##### Critical operational thresholds

The integrated sensitivity framework reveals operational trends and formulation strategies under the dataset’s average conditions. These thresholds represent general behavioral patterns derived from partial dependence analysis, where parameter responses are computed while other features remain at typical values; specific values may shift under different formulation or reservoir contexts.

Permeability Accessibility Window: Analysis revealed a critical low-permeability transition (Sobol S₁ = 0.192, SHAP = 9.1°) where nano-cEOR effectiveness diminishes sharply. Below ~ 0.1 mD, CA responses plateau regardless of formulation, reconciling conflicting reports where failures occurred in ultra-tight rocks (K < 0.5 mD)^55,81^ while successes emerged in moderate-permeability substrates (K ≥ 5 mD)^42,82^. The permeability-NPs size interaction **(**Fig. 20**)** demonstrates size-selective accessibility: tighter formations favor smaller particles (< 20 nm), while higher-permeability rocks accommodate larger sizes (50–60 nm), enabling permeability-contingent formulation strategies^79^ (Ayatollahi et al. 2019).

Salinity Optimization Window: Non-monotonic salinity dependence emerges, with optimal performance in the moderate range and degradation at hypersaline conditions. This U-shaped response validates competing electrical double layer mechanisms; moderate salinity compresses Debye length enabling NPs-surface approach while divalent cations bridge negatively charged NPs to carbonates^83^, whereas excessive compression triggers aggregation^84^, suggesting salinity management may be required for offshore applications (Ayatollahi et al., 2019).

NPs-Polymer Synergy Ratio: Synergistic NPs to polymer interactions exhibit optimal performance specified in Sect. 3.3.4. At lower polymer loadings, minimal benefits occur, whereas at higher concentrations, polymers provide additional CA reduction (~ 6–8°) through steric stabilization preventing aggregation^85^. Unlike surfactants, polymers adsorb on NPs surfaces maintaining interparticle separation where electrostatic mechanisms become insufficient.

##### Rock type conditional optimization

Lithology-Dependent NPs Selection: ZrO_2_ achieves 21° reduction on carbonates versus Fe_3_O_4_’s 13° reduction on sandstones, representing performance inversion between lithologies. This correlates with surface charge magnitude (ZrO_2_ zeta potential =  − 45 to − 50 mV^80^) but MgO’s enhanced dolomite effectiveness (4th rank versus 8th overall) despite moderate charge (zeta potential ≈ − 22 mV) suggests lattice matching between Mg2⁺ in NPs structure and Mg2⁺-rich dolomite (CaMg(CO₃)₂). This “chemical recognition” mechanism, documented in mineral flotation^86^ but not previously in nano-cEOR, enables cation-matched design strategies (CaO for calcite, MgO for dolomite) exploiting specific adsorption beyond generic electrostatics.

Chemical Additive Rock-Dependency: Biosurfactants demonstrate robust performance across lithologies due to amphoteric functionality enabling adaptive electrostatic orientation^87^, while synthetic surfactants show rock-specific behavior validating charge-mediated adsorption. Anionic surfactants achieve 12° on positively charged carbonates but only 7° on negatively charged sandstone, with cationic surfactants inverting this trend. This provides simple selection rules: measure reservoir mineral’s zeta potential, and select surfactants with opposite-charge to maximize electrostatically driven adsorption.

##### Reconciling sobol-SHAP divergence

NPs concentration’s apparent contradiction negligible Sobol S1 (0.005) versus substantial SHAP importance (6.8°, rank 5)—illuminates critical differences between variance-based and attribution-based methods. In our dataset, 82% of samples concentrate in 0.05–0.30 wt% (low variance), while 18% span 0.30–1.50 wt% exhibiting dramatic non-linear responses where high concentrations with polymer produce 20–25° reductions but without polymer trigger aggregation collapse^74,75^. This “sparse influence” phenomenon indicates NPs concentration is critical for formulation optimization (SHAP identifies) but not primary dataset variability driver (Sobol identifies). Practical implications: field applications constraining concentrations to 0.1–0.3 wt% offer limited optimization gains (2–3°), while high-value applications justifying 0.5–1.0 wt% require co-optimization with polymer to access the synergistic window. This validates multi-method approaches where Sobol guides data collection priorities while SHAP guides operational optimization^76,78^.

#### Benchmarking against the state-of-the-art

Recent advances in ML for CA and wettability prediction, particularly in Nano-assisted and energy-relevant systems, have produced promising but variable results. Reported performance typically spans RMSE of 5.7–12.0°, MAE below 5.0°, and validation or testing R^2^ values in the range of 0.82–0.92, as seen in XGBR and GBR models applied to both polymeric and geologic settings. For example, a recent XGBR based model reported RMSE of 5.7°, MAE of 3.7°, and R^2^ of 0.86 on the prediction of water CA for solid polymers, using CV but without an independent external validation set^38^.

In contrast, the present study achieves validation RMSE of 9.24°, MAE of 5.87°, and R^2^ of 0.95 using XGBR on a uniquely curated and externally validated nano-cEOR dataset, thereby matching or exceeding the best of existing literature while also offering several crucial enhancements. Most prior studies either optimize models on combined train/test folds or rely exclusively on internal CV^38^, whereas this study approach supplements these standard measures with a reserved validation split, mirroring real-world deployment scenarios and ensuring robust out-of-sample generalization.

Beyond predictive accuracy, this work establishes new standards in ML interpretability for EOR through comprehensive sensitivity frameworks absent in prior implementations. While recent studies incorporate feature importance typically single-method permutation^79^ or tree-based Gini indices^78^ our integrated Sobol-SHAP-PDP approach represents the most rigorous sensitivity framework in petroleum ML literature. Zhuang et al.^76^ applied SHAP to CO₂-EOR but limited analysis to rankings without interaction quantification. Yahya et al.^77^ employed Sobol for recovery factor prediction yet omitted categorical variables and conditional stratification. This study framework uniquely identifies operational thresholds providing formulation guidelines suitable for experimental validation.

The rock-type conditional sensitivity framework (Figs. 21 and 22) introduces methodological innovation unreported in CA literature. Traditional aggregate importance rankings^33^ mask lithology-dependent optimization opportunities. Our stratified analysis exposes inverted NPs selection between carbonate and sandstone reservoirs,insights unattainable through global metrics. This physics-informed conditional approach aligns with emerging reservoir ML paradigms^88^ encoding mineralogical surface chemistry into interpretation.

The Sobol-SHAP discrepancy reconciliation addresses a persistent methodological gap where conflicting metrics are dismissed as artifacts^75^. Demonstrating variance decomposition guides experimental design while marginal attribution drives operational optimization establishes a dual-purpose sensitivity blueprint applicable across reservoir engineering contexts.

Importantly, our benchmarking strategy extends beyond core performance metrics. This study provides additional diagnostic analytics including residual statistics (mean, standard deviation, min/max), generalization gap quantification, and symmetry analysis to quantitatively assess prediction bias, uncertainty, and overfitting risks. This multidimensional evaluation framework provides transparency not only in model accuracy, but also in calibration and reliability, thereby setting a new benchmark for methodological rigor and practical readiness in CA model reporting.

Together, these contributions position this workflow as a reference implementation for nano-cEOR wettability prediction. The integration of (1) superior generalization (R^2^ = 0.95 vs. literature 0.82 – 0.92), (2) multi-method sensitivity revealing thresholds and interactions, (3) conditional optimization for reservoir-specific formulation, and (4) diagnostic transparency for deployment readiness positions this framework as a promising tool for field-scale optimization pending experimental validation before field-applicable decision as demonstrated in 2025 peer-reviewed literature (Supplementary Material S2, Table 2).

#### Advantages, limitations, and field deployment considerations

The principal advantages of the proposed framework include: (1) Comprehensiveness of input descriptors; the framework is, to the authors’ knowledge, the first ML model for CA prediction to simultaneously incorporate NP-specific descriptors (type, concentration, size, functionalization) alongside standard reservoir and fluid properties, directly addressing the physical mechanisms unique to nano-cEOR; (2) Multi-method sensitivity architecture; the integrated Sobol–SHAP–PDP approach provides cross-validated, physically interpretable operational thresholds that are directly actionable in field formulation design, rather than abstract feature importance rankings; (3) External validation rigor; the use of three independent data partitions (training, testing, validation) plus tenfold nested cross-validation satisfies the highest standards of ML validation for experimental datasets, providing unbiased generalization estimates; (4) Rock-type stratification; lithology-specific formulation guidelines represent a practical advance over aggregate models, enabling reservoir engineers to tailor NP selection based on mineralogy; and (5) Computational efficiency; the trained XGBR model generates CA predictions in milliseconds, compared to hours of experimental measurement, enabling high-throughput nanofluid screening.

To guide future research, key limitations must be acknowledged. The model is trained on literature-aggregated data, introducing potential latent experimental inconsistencies (e.g., measurement techniques, surface preparation). Additionally, predictive capability is constrained by available features; parameters known to affect wettability such as surface roughness^15^ or pore-scale geometry^89^ are rarely reported and thus excluded, likely contributing to irreducible prediction error. CA prediction alone does not account for injectivity loss, NP retention, or long-term formation damage critical considerations for field viability that require complementary displacement simulation.

From an industry deployment perspective, the proposed framework is designed for integration into nanofluid screening workflows at the pre-pilot laboratory stage. A practitioner would input reservoir characterization data (rock type, permeability, porosity, formation water salinity, reservoir temperature) alongside candidate nanofluid formulation parameters (NP type, concentration, and size; chemical additive type and concentration) to rapidly obtain a CA prediction with associated uncertainty bounds. The sensitivity-derived thresholds (minimum permeability 0.1 mD, salinity window 30,000–80,000 ppm, NP:chemical ratio 1:1 to 1.5:1) provide direct screening criteria to reject unsuitable candidates without laboratory measurement. Deployment in commercial reservoir simulators would require coupling the ML CA predictor with relative permeability upscaling routines, and integration with uncertainty quantification tools (e.g., Monte Carlo ensemble prediction) to propagate input measurement uncertainty into CA confidence intervals. Validation through field pilot testing under representative reservoir conditions remains the critical final step before full operational deployment.

## Future Directions, Summary and conclusions

Based on the findings and limitations identified in this study, the following future research directions are recommended:

1. Experimental Validation: The rock-type conditional framework enables in silico identification of optimal formulations for specific lithologies (e.g., ZrO_2_ 0.3 wt% + biosurfactant 0.1 wt% at 50,000 ppm for carbonates; Fe_3_O_4_ 0.2 wt% + cationic surfactant 0.1 wt% at 5,000 ppm for sandstones) within identified operational windows (permeability > 0.1 mD, salinity < 100,000 ppm, NPs to polymer ratio from 1 to 1 up to 1.5 to 1), requiring targeted validation experiments.
2. Coupled interfacial teansion (IFT)-CA Modeling: Integrating this CA model with IFT prediction frameworks would create a comprehensive multi-output EOR screening tool incorporating NPs-polymer synergies and salinity-dependent threshold behaviors for modeling coupled wettability-interfacial phenomena^58,59,90^.
3. Physics-Informed Architectures: Physics-Informed Neural Networks (PINNs) could embed governing laws (Young’s Equation^91^), DLVO theory, ion bridging mechanisms, and permeability-accessibility constraints as soft loss function constraints, ensuring physically consistent predictions.
4. Threshold-Guided Sampling: Identified thresholds (0.1 mD permeability limit, 30,000–80,000 ppm optimal salinity, 100,000 ppm aggregation onset) should guide strategic oversampling within critical transition zones (0.1–3 mD, 80,000–120,000 ppm) where nonlinearity is most pronounced, improving accuracy in high-impact regions.
5. Transfer Learning: Given optimal NPs selection inversion between lithologies, transfer learning could leverage knowledge from data-rich reservoirs (limestone) to predict performance in data-scarce systems (dolomite, mixed mineralogy), reducing experimental burden.

This study establishes a validated ML framework for CA prediction in nano-cEOR systems, addressing critical gaps in predictive wettability modeling through integration of NPs-specific descriptors and physics-informed sensitivity analysis. Key findings include:

- Superior predictive performance: The XGBR model achieved validation RMSE of 9.24°, MAE of 5.87°, R^2^ of 0.953, and MAPE of 13.6%, with near-zero bias (mean residual: − 0.04°) and controlled generalization (138% gap), outperforming all competing algorithms including hybrid ANN-RF and matching or exceeding state-of-the-art benchmarks while providing full external validation on reserved test data.
- Actionable operational thresholds: Comprehensive multi-method sensitivity analysis (Sobol-SHAP-PDP integration) identified experimentally testable formulation guidelines absent from prior ML-EOR implementations. Minimum permeability of 0.1 mD for nano-cEOR viability, optimal salinity window of 30,000–80,000 ppm avoiding aggregation beyond 100,000 ppm, and NPs to polymer synergistic ratio of 1 to 1 up to 1.5 to 1 achieving 6–8° additional CA reduction.
- Rock-type conditional optimization: Stratified sensitivity analysis revealed lithology-dependent formulation strategies impossible to derive from aggregate feature importance rankings, with optimal NPs selection inverting between carbonates (ZrO₂ achieves 21° reduction) and sandstones (Fe₃O₄ achieves 13° reduction), and chemical additives exhibiting strong mineralogical dependence (biosurfactants achieves18° on carbonates; cationic surfactants achieves 11° on sandstones).
- Methodological innovation: Reconciliation of variance-based (Sobol) and attribution-based (SHAP) methods established a dual-purpose sensitivity blueprint where variance decomposition guides experimental design priorities while marginal attribution drives operational optimization, addressing persistent gaps where conflicting metrics are dismissed as artifacts.

The novelty lies in advancing nano-cEOR ML toward field-applicable decision support pending validation through integration of predictive accuracy (R^2 ^> 0.95), sensitivity-derived operational thresholds, and reservoir-specific formulation guidelines. Future validation through field pilots and extension to multicomponent nanofluid systems should further enhance deployment readiness.

## Supplementary Information

Supplementary Information 1.

Supplementary Information 2.

Supplementary Information 3.

Supplementary Information 4.

## Data Availability

The complete experimental dataset (n = 418) underlying this study, including all input features, source references, and experimentally measured contact angle values, is provided as Supplementary Material S3. The dataset is organized into two partitions: the training set (n = 314, Sheet 1) and the combined testing and validation set (n = 104, Sheet 2). Predicted contact angle values from the three highest-performing models (GBR, XGBR, and ANN-RF Hybrid), alongside true experimental values in both normalized and degree form, are provided for all 418 data points in Supplementary Material S4. These materials are intended to facilitate independent verification of all reported performance metrics, support reproducibility of the sensitivity analysis, and enable further use and extension of the dataset and models by the broader research community.

## Abbreviations

**ANN**: Artificial neural network
CA: Contact angle
**CDF**: Cumulative distribution function
**cEOR**: Chemical enhanced oil recovery
CI: Confidence interval
CV: Cross-validation
EOR: Enhanced oil recovery
GBR: Gradient boosting regressor
IFT: Interfacial tension
**LR**: Linear regression
MAE: Mean absolute error
**MAPE**: Mean absolute percentage error
ML: Machine learning
MSE: Mean square error
nano-cEOR: Nano-assisted chemical enhanced oil recovery
NPs: Nanoparticles
OHE: One-hot encoding
PDP: Partial dependence plots
PIML: Physics-informed machine learning
R^2^: Coefficient of determination
RF: Random forest
RMSE: Root mean squared error
S1: First-order effect
**ST**: Total-order effect
SG: Specific gravity
Tanh: Hyperbolic tangent
WA: Wettability alteration
XGBR: Extreme gradient boosting regressor
θ: Contact angle (degrees, °)
xi: Value of input feature i
x’: Min–Max normalized feature value
yi: Experimentally measured CA for data point i (°)
ŷi: Model-predicted CA for data point i (°)
ȳ: Mean of experimentally measured CA values (°)
μ: Population or sample mean
σ: Standard deviation
n, N: Number of data points / samples
K: Permeability (millidarcy, mD)
r: Pearson or Spearman correlation coefficient
