# Supplementary material for: A robust machine learning framework for predicting contact angle in nano-assisted chemical EOR
Source: Sci Rep. 2026 May 8;16:14676. doi: 10.1038/s41598-026-48016-1 (PMC13156270; doi:10.1038/s41598-026-48016-1)
Supplement: Supplementary file 1 — Supplementary Information 1. [file 41598_2026_48016_MOESM1_ESM.pdf]

A Robust Machine Learning Framework for Predicting Contact Angle in Nano-Assisted Chemical EOR

Youssef E. Kandiel[1*], Omar Mahmoud[2], Ahmed Farid Ibrahim[3,4]

[1]Department of Petroleum and Energy Engineering, School of Sciences and Engineering, American University in Cairo (AUC), Cairo, Egypt
[2] Department of Chemical and Petroleum Engineering, College of Engineering and Physical Sciences, Khalifa University, 127788 Abu Dhabi, United Arab Emirates
[3] Department of Petroleum Engineering and Geosciences, King Fahd University of Petroleum & Minerals, Dhahran, 31261, Saudi Arabia
[4] Center for Integrative Petroleum Research, King Fahd University of Petroleum & Minerals, 31261 Dhahran, Saudi Arabia
[*]Corresponding Author: Youssef E. Kandiel: youssef.kandiel@aucegypt.edu; ORCID number: 0000-0001-7422-2149

$$x' = \frac{x - \min(x)}{\max(x) - \min(x)} \quad (1)$$

where x is the feature value after logarithmic transformation and x ' is the normalized value.

$$R = \frac{\left[\frac{\sum(x_i - \mu_x)\ (y_i - \mu_y)}{n-1}\right]}{\sigma_x\ \sigma_y} \quad (2)$$

where R is the correlation coefficient for the output parameter (output =CA) and input parameters, x_i is the independent feature which involves all input parameters, y_i is the dependent parameter (CA). $\mu_x, \mu_y, and\ \sigma_x, \sigma_y$ are the mean and the standard deviation for the input and the output parameters, respectively.

Model performance was quantified using four standard statistical metrics:

1. **R-squared (R^2):** The coefficient of determination, which measures the proportion of the variance in the output variable that is predictable from the input features. A value of 1.0 indicates a perfect fit calculated as in Equation 3.

$$R^2 = 1 - \frac{\sum_{i=1}^{N}(y_i - \hat{y}_i)^2}{\sum_{i=1}^{N}(y_i - \bar{y})^2} \quad (3)$$

2. **Mean Absolute Error (MAE):** The average of the absolute differences between the predicted and actual values. This metric is highly interpretable as it represents the average error in the same units as the target (degrees) as in Equation 4.

$$MAE = \frac{1}{N}\sum_{i=1}^{N}|y_i - \hat{y}_i| \quad (4)$$

3. **Root Mean Squared Error (RMSE):** The square root of the average of squared differences. This metric penalizes larger errors more heavily than MAE, calculated as in Equation 5.

$$RMSE = \sqrt{\frac{1}{N}\sum_{i=1}^{N}(y_i - \hat{y}_i)^2} \quad (5)$$

4. **Mean Absolute Percentage Error (MAPE)**

Mean Absolute Percentage Error (MAPE) is a widely adopted performance metric for evaluating regression models, quantified as the mean of absolute relative errors between predicted and actual values, expressed as a percentage to facilitate intuitive interpretation of prediction accuracy, Equation 6. This metric is particularly valuable when assessing relative variations rather than absolute deviations, making it especially useful for comparing model performance across datasets with different scales, such as EOR applications where reservoir parameters exhibit high variability (Glänzer et al., 2024).

$$MAPE = \frac{100}{n}\sum_{i=1}^{n}\left|\frac{y_i - \hat{y}_i}{y_i}\right| \quad (6)$$

All six machine learning algorithms were further evaluated using comprehensive performance metrics including prediction consistency (residual standard deviation) Equation 7, systematic bias (mean residual) Equation 8, worst-case performance (min/max residuals) Equation 9, and generalization capability (training-validation gap) Equation 10.

$$Residual\ St.Dev. = \sqrt{\frac{\sum(Actual - Predicted)^2}{n}} \quad (7)$$

$$Mean\ Residual = \frac{\sum(Actual - Predicted)^2}{n} \quad (8)$$

$$\frac{\text{min}}{\text{max}}\text{residuals} = The\ extreme\ bounds\ of\ prediction\ errors\ -\ worstcase\ scenarios \quad (9)$$

$$\text{Generalization Capability} = \frac{Val\ RMSE - Train\ RMSE}{Train\ RMSE} * 100\% \quad (10)$$
