# Supplementary material for: A robust machine learning framework for predicting contact angle in nano-assisted chemical EOR
Source: Sci Rep. 2026 May 8;16:14676. doi: 10.1038/s41598-026-48016-1 (PMC13156270; doi:10.1038/s41598-026-48016-1)
Supplement: Supplementary file 2 — Supplementary Information 2. [file 41598_2026_48016_MOESM2_ESM.pdf]

A Robust Machine Learning Framework for Predicting Contact Angle in Nano-Assisted Chemical EOR

Youssef E. Kandiel[1*], Omar Mahmoud[2], Ahmed Farid Ibrahim[3,4]

[1]Department of Petroleum and Energy Engineering, School of Sciences and Engineering, American University in Cairo (AUC), Cairo, Egypt
[2] Department of Chemical and Petroleum Engineering, College of Engineering and Physical Sciences, Khalifa University, 127788 Abu Dhabi, United Arab Emirates
[3] Department of Petroleum Engineering and Geosciences, King Fahd University of Petroleum & Minerals, Dhahran, 31261, Saudi Arabia
[4] Center for Integrative Petroleum Research, King Fahd University of Petroleum & Minerals, 31261 Dhahran, Saudi Arabia
[*]Corresponding Author: Youssef E. Kandiel: youssef.kandiel@aucegypt.edu; ORCID number: 0000-0001-7422-2149

Table 1. Hyperparameter optimization of various ML algorithms

Algorithm	Hyperparameter	Available Options	Optimum Option
RF	n_estimators	100, 150, 200, 300	100
	Maximum depth	5, 10, 20, 30	20
	Maximum features	auto, sqrt, log2	log2
	Bootstrap	True, False	FALSE
	Min samples split	2, 5, 10	2
	Min samples leaf	1, 2, 4	1
XGBR	n_estimators	100, 150, 200, 300	150
	Maximum depth	3, 5, 7, 13, 20, 25	13
	Learning rate	0.01, 0.05, 0.1, 0.3	0.3
	Subsample	0.7, 0.8, 1.0	0.8
	Colsample_bytree	0.7, 0.8, 1.0	0.8
	Gamma	0, 0.1, 0.2	0
	Reg alpha	0, 0.01, 0.1	0.1
	Reg lambda	1, 1.5, 2.0	2
GBR	n_estimators	100, 200, 300	300
	Learning rate	0.01, 0.05, 0.1	0.05
	Maximum depth	3, 5, 7, 13, 15, 20, 25	25
	Subsample	0.7, 0.8, 1.0	0.7
	Min samples split	2, 5, 10	5
	Min samples leaf	1, 2, 4	2
ANN	Hidden layers	4	4

	Neurons per layer	265	265
	Activation function	relu, tanh, sigmoid	tanh
	Learning rate	0.0001, 0.001, 0.01	0.001
	Optimizer	Adam, SGD, RMSprop	Adam
LR	Regularization	None, Ridge, Lasso, ElasticNet	Ridge
	Alpha	0.001, 0.01, 0.1, 1.0, 10.0, 100.0	1
	Fit intercept	True, False	TRUE
	Solver	auto, svd, lsqr, saga	auto

Table 2. Benchmarking of this study against existing literature.

Study	System	Algorithm	Validation Strategy	Best R^2	RMSE (°)	MAE (°)	Sensitivity Method	Operational Outputs
Sena et al. (2025)	Polymer surfaces	XGBR	CV only (no external validation)	0.86	5.7	3.7	None reported	None
Zhuang et al. (2025)	CO_2-EOR	Ensemble	Train/test split	0.88	N/R	N/R	SHAP (rankings only)	Feature rankings
Yahya et al. (2025)	Recovery factor	RF	CV only	0.92	N/R	N/R	Sobol (continuous only)	Variance decomposition
Hajibolouri & Shafiei (2025)	H_2 storage	GBR	Train/test split	0.89	8.2	N/R	Permutation importance	Aggregate rankings
Vo Thanh et al. (2023)	Sandstone CA	ANN	CV only	0.87	10.5	N/R	Gini index	None
Wahyudi et al. (2025)	Carbonate perm.	Hybrid DL	Train/test/validation	0.91	N/R	N/R	None	None
This Study	nEOR (multi-lithology)	XGBR + ANN-RF	Train/test/validation + 10-fold CV	0.95	9.24	5.87	Sobol + SHAP + PDP + conditional stratification	Permeability threshold (0.1 mD), salinity window (30–80k ppm), NP:polymer ratio (1:1–1.5:1), rock-specific NP selection
